# SETD1A protects from senescence through regulation of the mitotic gene expression program

**DOI:** 10.1038/s41467-019-10786-w

**Published:** 2019-06-28

**Authors:** Ken Tajima, Satoru Matsuda, Toshifumi Yae, Benjamin J. Drapkin, Robert Morris, Myriam Boukhali, Kira Niederhoffer, Valentine Comaills, Taronish Dubash, Linda Nieman, Hongshan Guo, Neelima K. C. Magnus, Nick Dyson, Toshihiro Shioda, Wilhelm Haas, Daniel A. Haber, Shyamala Maheswaran

**Affiliations:** 1 Massachusetts General Hospital Cancer Center, Harvard Medical School, Charlestown, MA 02129 USA; 2 Department of Surgery, Massachusetts General Hospital, Harvard Medical School, Boston, MA 02114 USA; 3 Division of Medical Oncology, Massachusetts General Hospital, Harvard Medical School, Boston, MA 02114 USA; 4 Howard Hughes Medical Institute, Chevy Chase, MD 20815 USA; 5 Present Address: Department of Respiratory Medicine, Juntendo University School of Medicine, Tokyo, 113-8421 Japan; 6 Present Address: Department of Surgery, Keio University School of Medicine, Tokyo, 160-8582 Japan

**Keywords:** Cell division, Mitosis

## Abstract

*SETD1A*, a *Set1*/COMPASS family member maintaining histone-H3-lysine-4 (H3K4) methylation on transcriptionally active promoters, is overexpressed in breast cancer. Here, we show that *SETD1A* supports mitotic processes and consequentially, its knockdown induces senescence. SETD1A, through promoter H3K4 methylation, regulates several genes orchestrating mitosis and DNA-damage responses, and its depletion causes chromosome misalignment and segregation defects. Cell cycle arrest in SETD1A knockdown senescent cells is independent of mutations in *p53*, *RB* and *p16*, known senescence mediators; instead, it is sustained through transcriptional suppression of SKP2, which degrades p27 and p21. Rare cells escaping senescence by restoring SKP2 expression display genomic instability. In > 200 cancer cell lines and in primary circulating tumor cells, SETD1A expression correlates with genes promoting mitosis and cell cycle suggesting a broad role in suppressing senescence induced by aberrant mitosis. Thus, *SETD1A* is essential to maintain mitosis and proliferation and its suppression unleashes the tumor suppressive effects of senescence.

## Abstract (web summary)

SETD1A, a histone H3K4 methyltransferase that promotes gene expression, is required for embryonic development. Here, the authors show that SETD1A regulates the expression of mitotic genes and that SETD1A suppression induces senescence.

## Introduction

Senescence is a prolonged state of growth arrest induced by a variety of stimuli including oncogene activation, oxidative and metabolic stress and mitotic aberrations^1,2^. It is a physiological process involved in aging and age-related diseases^3,4^ and is an important tumor suppressor mechanism in premalignant tumors. A subset of these preneoplastic lesions including benign nevi, lung adenomas, and prostatic intra-epithelial neoplasia eventually override this safeguard mechanism through the loss of *p53*, *RB*, *INK4a* and *ARF* and progress to malignant tumors^5–8^. Senescence is also an alternative cellular response to chemo- and radiation-therapies, which induce extensive DNA damage. A minor fraction of therapy-induced senescent cells (TIS) override growth arrest through acquisition of stem-like properties^9^ and unstable genomes^10^ and the cells escaping senescence exhibit highly drug resistant phenotypes^11,12^. However, the mechanisms that underlie senescence, particularly in cancer cells that have already inactivated *RB* and *p53* signaling are not defined, and these may offer the potential for modulating this tumor suppressive pathway.

In a shRNA screen of chromatin modifiers^13^, we identified SETD1A as one of the most potent regulators of key genes driving mitosis. *SETD1A* encodes a highly conserved member of the multi-subunit *Set1*/COMPASS complex^14^, whose functional paralogues, *Set1* and *dSETD1* in yeast and Drosophila, respectively, are critical to maintain proliferation and viability^15–19^. Gene knockdown of *Setd1a* in mice causes severe proliferative defects during embryonic development^20^, suggesting that this function of SETD1A is evolutionarily conserved. Although the loss of SET proteins in multiple organisms causes extensive proliferative defects, their involvement in the maintenance of overall H3K4 methylation under these conditions has precluded our understanding of the specific molecular mechanisms underlying these functional defects.

Here we show that by regulating H3K4 methylation on the promoters of mitotic genes, SETD1A maintains the integrity of the mitotic process in cells. As a result, SETD1A knockdown, independent of the *p53* and *RB* status in cells, leads to severe mitotic defects and senescence, suggesting that SETD1A plays a pivotal role in maintaining the balance between multiple cellular processes involved in cellular fitness.

## Results

### Suppression of SETD1A induces senescence

**Fig. 1:**
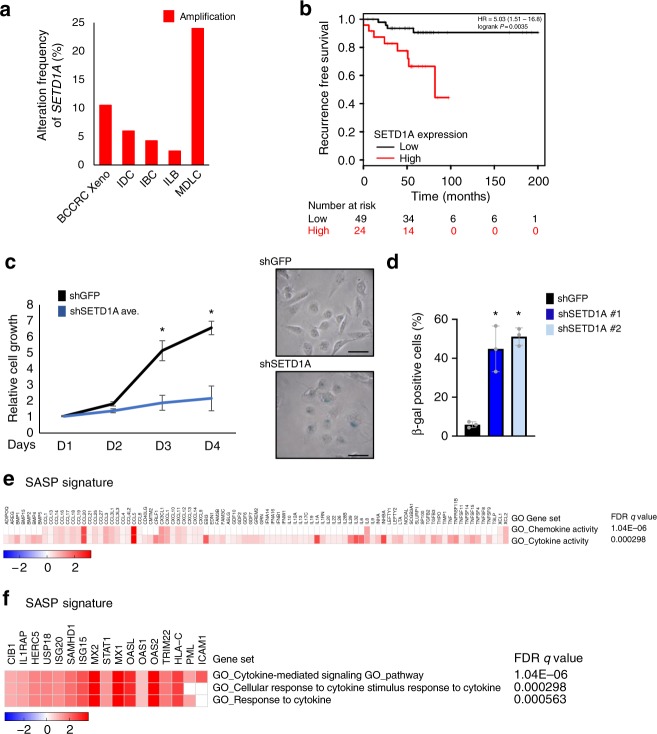
SETD1A expression protects cells from senescence. **a** *SETD1A* is amplified in breast cancer. Publicly available data from 935 breast cancers (http://www.cbioportal.org/) was evaluated for *SETD1A* gene amplification. The frequency of amplification in mixed ductal and lobular (MDLC), invasive ductal carcinoma (IDC) and invasive lobular carcinoma (ILB) of the breast is shown. IBC represents invasive breast carcinoma. Clonal evolution of breast cancer patient derived xenografts in mice, studied at single-cell resolution^21^, shows that 24% of the resulting tumors exhibit *SETD1A* gene amplification (BCCRC-Xeno). Source data are provided as a Source Data file. **b** Kaplan–Meier analysis was used to plot the overall survival of hormone receptor positive breast cancer patients with high (upper tertile) and low SETD1A expression. *p* value was calculated using log-rank test (Logrank p = 0.0035; HR = 5.03 (1.51–16.8). **c** SETD1A depletion induces senescence. Left: Relative proliferation of MDA-MB-231 cells infected with shGFP and shSETD1A. shSETD1A_av_ represents the mean of cells infected with two different shSETD1A constructs. Data from three independent experiments are presented as Mean + SD; *p < 0.05 by two-tailed unpaired Student’s *t* test. Source data are provided as a Source Data file. Right: Images of ß-gal stained control (shGFP) and SETD1A-KD (shSETD1A) MDA-MB-231 cells are shown. The scale bar represents 50 µm. **d** Bar graph shows the percentage of ß-gal positive cells in MDA-MB-231 cultures infected with shSETD1A and shGFP. shSETD1A_av_ represents the mean of cells infected with two different shSETD1A constructs. Data from three independent experiments are presented as Mean + SD; *p < 0.05 by two-tailed unpaired Student’s t test. Source data are provided as a Source Data file. **e** Senescence-associated secretory phenotype (SASP) in SETD1A-KD cells. RNAs showing log2 fold change > 1(FDR *q* value > 10%) in both SETD1A-KD (compared with shGFP) MDA-MB-231 and A549 cells were analyzed by GSEA for the enrichment of cytokine and chemokine activity. Genes contributing to the enrichment of each pathway and FDR q-values are provided. **f** Proteomic analysis of SASP in SETD1A-KD cells. Proteins showing log2 fold change > 1(FDR *q* value > 10%) in both SETD1A-KD (compared with shGFP) MDA-MB-231 and A549 cells were analyzed by GSEA for the enrichment of cytokine and chemokine activity. The fold induction of the genes contributing to the enrichment of each pathway and FDR q-values are provided

The overexpression of SETD1A in multiple tumor types^13^ suggests an aberrant adaptation of this chromatin regulator in cancer cells. Analysis of publicly available data from 935 breast cancers (http://www.cbioportal.org) shows that SETD1A is amplified in 7% of cases and in 12% of mixed ductal and lobular breast carcinomas (MDLC). Studies of clonal evolution in breast cancer patient-derived xenografts in mice analyzed at single-cell resolution^21^ show that 24% of the resulting tumors exhibit SETD1A gene amplification (Fig. 1a). Furthermore, overexpression of SETD1A in breast cancers is associated with poor clinical outcome (Fig. 1b; Logrank *P* = 0.0035; HR = 5.03 (1.51–16.8)), suggesting that it confers a growth advantage in multiple tumor settings.

To study the role of *SETD1A* in cancer, we suppressed its expression in cancer cells. Remarkably, knockdown of SETD1A (SETD1A-KD) suppresses proliferation and triggers prompt (72 h) and massive cellular senescence, with very large cells expressing characteristic ß-galactosidase (ß-gal) activity (Fig. 1c, d; Supplementary Fig. 1a, 1b, Data from three independent experiments are presented as Mean + SD; *p < 0.05 by two-tailed unpaired Student’s t test). Suppressing SETD1A expression in the immortalized but non-tumorigenic human mammary epithelial cell line, MCF10A, also leads to increased senescence compared with shGFP expressing control cells (Supplementary Fig. 1c, Data from two independent experiments are presented as Mean + SD; *p < 0.05 by two-tailed unpaired Student’s t test). In addition to increasing the expression of the senescence core signature^22^ (Supplementary Fig 1d), SETD1A-KD in cells increases cytokine and chemokine activity consistent with the senescence-associated secretory phenotype (SASP), both at the RNA and protein levels (Fig. 1e, f).

**Fig. 2:**
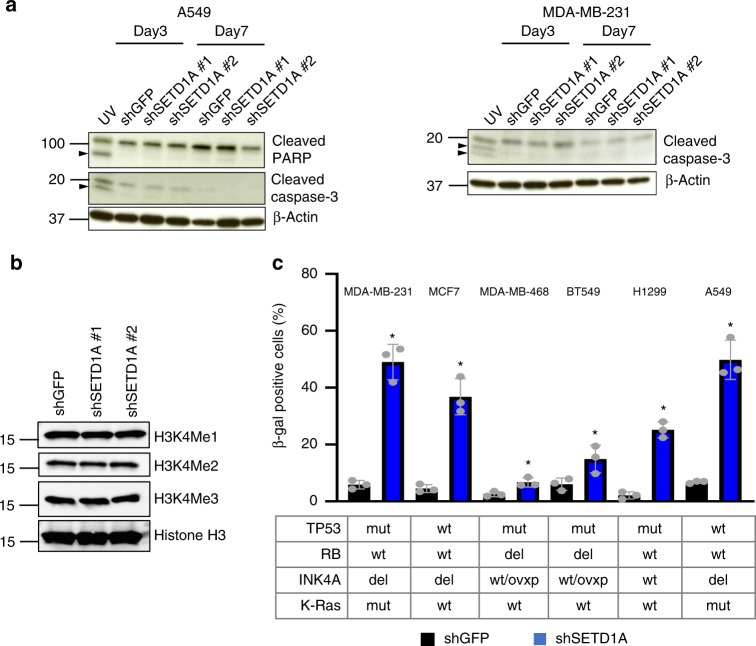
SETD1A-KD induces senescence independent of *p53* and *RB* status. **a** SETD1A-KD does not induce apoptosis. Proteins were analyzed after 3 and 7 days of SETD1A-KD for the expression of cleaved caspase-3 (A549 and MDA-MB-231) and cleaved PARP (A549). Cells irradiated with UV are shown as positive controls and the cleaved caspase-3 and cleaved PARP fragments in the positive control are highlighted with arrows. ß-actin serves as the loading control. Source data are provided as a Source Data file. **b** SETD1A-KD does not change global H3K4 methylation. shGFP- and SETD1A-KD-MDA-MB-231 cells were analyzed for H3K4Me1, H3K4Me2, H3K4Me3 and total histone-3 protein expression. Source data are provided as a Source Data file. **c** Bar graphs show quantification of ß-gal positive cells (Mean + SD) in multiple breast cancer (MCF7, MDA-MB-468, BT549, MDA-MB-231) and lung cancer (HT1299, A549) cell lines infected with shSETD1A and shGFP. The mutational status of *p53*, *RB*, *p16* and *K-Ras* in these cell lines is shown below. wt wild type, del deletion, mut mutant, wt/ovxp wildtype or overexpressed. shSETD1A_av_ represents the mean of cells infected with two different shSETD1A constructs. Data from three independent experiments are presented as Mean + SD; *p < 0.05 by two-tailed unpaired Student’s *t* test. Source data are provided as a Source Data file

The cell cycle arrest resulting from SETD1A-KD (Supplementary Fig. 2a; Data from three independent experiments are presented as Mean + SD; *p < 0.05 by two-tailed unpaired Student’s *t* test) is consistent with cellular senescence, and it is not associated with apoptosis as seen by the absence of the caspase-3 and PARP cleavage markers of apoptosis, following 3- and 7-days of SETD1A-KD^23,24^ (Fig. 2a). Despite its developmental role in regulating global H3K4 methylation^20^, SETD1A*-*KD in cancer cells does not affect total H3K4 mono-, bi, or tri-methylation, suggesting that the senescence phenotype in these cells is limited to more specific targets (Fig. 2b). Induction of senescence following SETD1A-KD is observed in all cell lines tested (breast cancer, *N* = 4 cell lines; lung, *N* = 2 cell lines; colon, *N* = 7 cell lines), independent of the mutational status of *TP53, RB, K-Ras* and *INK4A*, known mediators of cellular senescence signals^1,2,25^ (Fig. 2c, Supplementary Fig. 2b; Data from three independent experiments are presented as Mean + SD; *p < 0.05 by two-tailed unpaired Student’s *t* test).

### SETD1A induces mitosis and DNA damage response genes

**Fig. 3:**
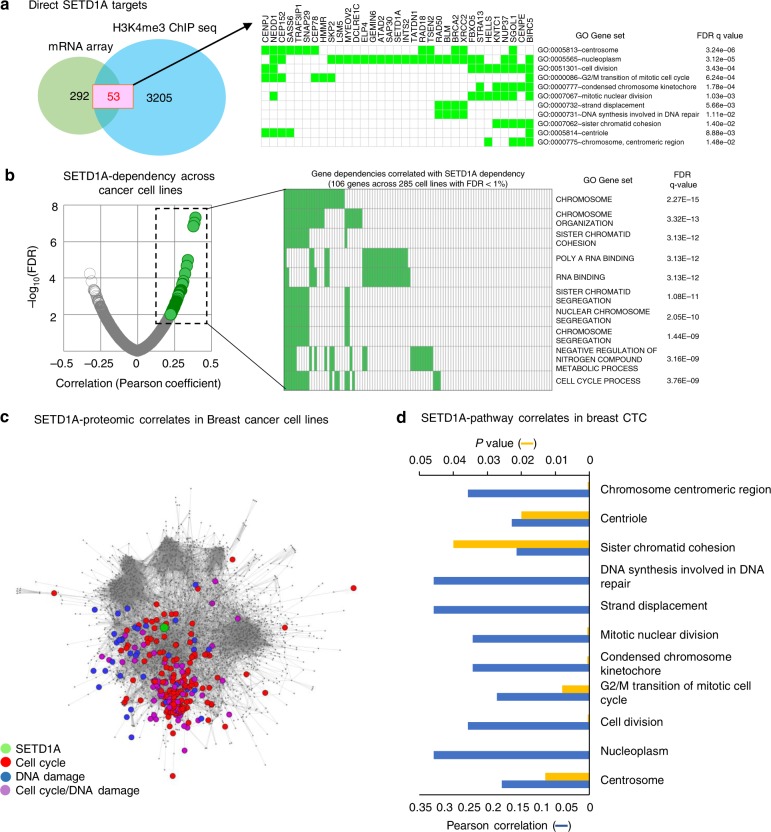
SETD1A regulates mitosis, cell cycle and DNA damage response genes. **a** Left: Transcriptome analysis of SETD1-KD A549 and MDA-MB-231 cells identified 345 genes suppressed across both cell lines (Supplementary Fig. 3a). shGFP-cells were used as control. Comparison of these 345 genes against 3258 H3K4 methylation marks suppressed in SETD1A-KD cells identified 53 direct SETD1A targets (Supplementary Fig. 3a). Right: The 53 SETD1A targets are enriched for targets involved in mitosis, cell cycle regulation and DNA damage response. Genes contributing to the enrichment of each pathway and FDR q-values are shown. **b** SETD1A-dependent cancer cells are sensitive to inactivation of genes involved in cell cycle and mitosis Left: 17,079 gene dependencies were ranked by correlation with SETD1A dependence across 285 cancer lines, as measured by Project Achilles (DEMETER v2.20.2 gene scores). Pearson coefficient vs. -log FDR is plotted for each gene (gray), and significant direct correlates are highlighted (green, FDR < 1%). Right: GSEA on the 106 gene dependencies best correlated with SETD1A dependence (black square in Fig. 2b on the left). Top 10 most significant overlaps, accounting for 57% of genes analyzed, are shown. 7/10 GO gene sets are associated with sister chromatid cohesion. **c** Analysis of high-throughput quantitative proteome data from 41 breast cancer lines shows that endogenous baseline SETD1A protein expression correlates with pathways involving mitosis, cell cycle and DNA damage responses. Cytoscape network map depicting proteins enriched by greater than log_2_(0.5) by quantitative MS (GSEA FDR ≤ 0.25; Nominal *p* value cutoff < 0.05). The heatmap displaying the hierarchical clustering of leading-edge proteins related to the processes indicated are shown in Supplementary Fig. 3d. The full set of gene enrichment results are provided in Supplementary table 1. **d** SETD1A expression in CTCs from metastatic breast cancer patients correlates with the expression of SETD1A-targets involved in cell cycle, mitosis and DNA damage responses. Graph shows the Pearson correlation coefficient between the leading edge-genes identified for each of the gene signatures shown in (**a**) and SETD1A expression in RNASeq data from single CTCs derived from breast cancer patients^29,30^. The *p* values for each pathway is provided. Source data are provided as a Source Data file

To dissect the potential mechanisms underlying SETD1A-KD senescence, we performed transcriptome analysis using two different cell types (breast cancer MDA-MB-231 and lung cancer A549), in which SETD1A-KD induces senescence (Fig. 1c, d, Supplementary Fig. 1b). Comparison of the transcriptome changes in control and SETD1A-KD cells across both cell lines identified 345 shared transcripts that were suppressed by multiple shRNA constructs (Supplementary Fig. 3a). To determine which of these transcripts are directly regulated through SETD1A-mediated changes in H3K4Me3 chromatin marks on their promoters, deposited through its methyl transferase activity^14^, we performed genome-wide H3K4Me3 ChIP-Sequencing in control and SETD1A-KD MDA-MB-231 cells. We identified 3258 loci with significant reduction in H3K4 trimethylation in cells, 42% of which resided within gene promoter regions (Supplementary Fig. 3b). Overlapping the transcriptional and chromatin changes induced by shRNA suppression of SETD1A nominated 53 genes as candidate direct targets regulated by changes in H3K4Me3 chromatin marks on their promoters (Fig. 3a, Supplementary Fig. 3c). As a third measure of functional significance, whole proteome analysis [(multiplexed mass spectrometry (MS)] using isobaric tandem mass tag (TMT) labeling^26,27^ of SETD1A-KD in both cell lines using two independent shRNA constructs, identified 33 of the 36 (92%) SETD1A targets detected by MS as being also reduced in protein expression (Supplementary Fig. 3d).

Gene Set Enrichment Analysis (GSEA) of the 53 consensus SETD1A targets identified pathways involved in mitosis and cell cycle regulation, and DNA-damage response as the top hits (FDR ≤ 0.25; Fig. 3a). Consistent with these findings, in the Broad Institute genome-wide shRNA screening database across 285 cancer cell lines (www.broadinstitute.org/achilles), the top 10 pathways correlated with SETD1A-KD (accounting for 57% of genes analyzed), correspond to cell cycle and mitosis, of which 7 gene sets are significantly associated with sister chromatid cohesion (Fig. 3b, Supplementary Fig. 4a). SETD1A expression is similarly associated with mitosis, cell cycle and DNA damage response pathways, as demonstrated by high-throughput quantitative proteome data using 41 breast cancer cell lines^28^ (Fig. 3c, Supplementary Fig. 4b). To define the extent to which the correlation between SETD1A expression and these cellular processes is relevant in clinical samples, we analyzed single-cell RNA-Sequencing data derived from primary patient-derived circulating tumor cells (CTCs) enriched from women with advanced breast cancer^29,30^, which also confirmed the correlation in expression between SETD1A and genes involved in mitosis and DNA damage repair (Fig. 3d). Taken all together, these findings show that SETD1A expression, consistent with its methyl transferase activity, by modulating H3K4Me3 marks on promoters, positively regulates the expression of a subset of genes required for execution of cell division. These chromatin-mediated transcriptional effects resulting from the changes in SETD1A-induced H3K4 methylation of promoters appear to differ from the role for SETD1A in mediating the survival of acute myeloid leukemia cells through a non-enzymatic function mediated through its interaction with cyclin K^31^.

### SETD1A-KD induces mitotic defects

**Fig. 4:**
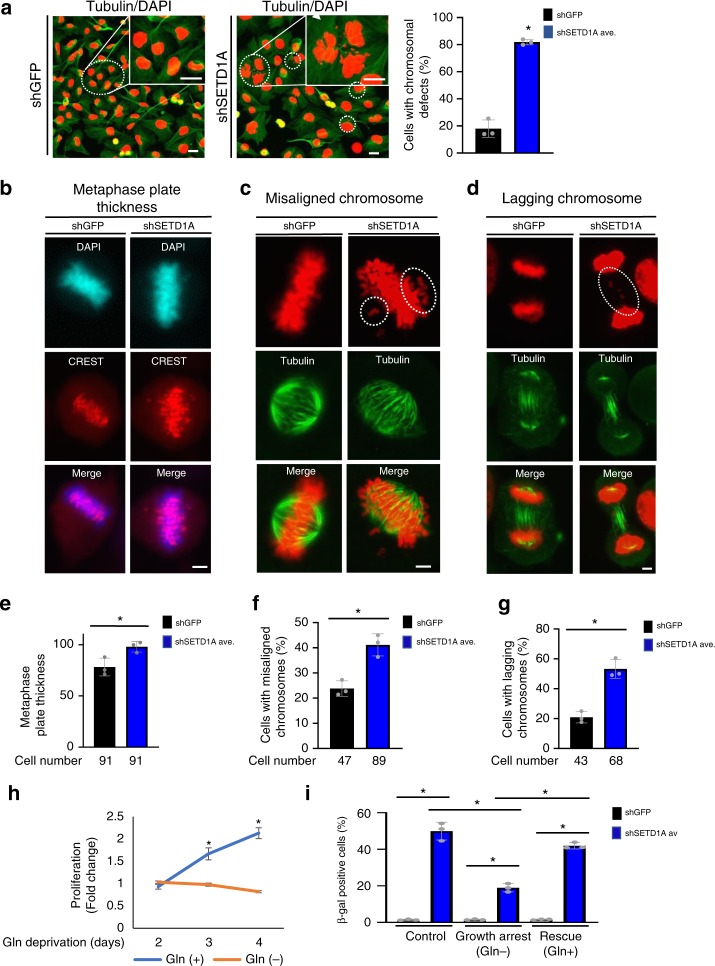
SETD1A expression is required for proper mitosis. **a** MDA-MB-231 cells were stained with an antibody against tubulin (green) and the nuclear stain, DAPI (red). Micronuclei resulting from chromosome segregation defects in SETD1A-KD cultures are highlighted with dashed circles. Scale bar represents 20 µm. Quantification of the fraction of cells with micronuclei/chromosome segregation defects are provided in the bar graph. Data from three independent experiments are presented as Mean + SD; *p < 0.05 by two-tailed unpaired Student’s *t* test. Source data are provided as a Source Data file. **b** shGFP- and shSETD1A-MDA-MB-231 cells were stained with antibodies against CREST protein to mark the centromeres (red). Nuclei were stained with DAPI (blue). Photomicrographs show poor alignment of chromosomes during metaphase increasing the plate thickness. The scale bar represents 5 µm. Source data are provided as a Source Data file. **c**, **d** shGFP- and shSETD1A-MDA-MB-231 cells were stained with antibodies against tubulin to mark the microtubules (green). Nuclei were stained with DAPI (red). Mitotic cells with mis-aligned chromosomes (**c**) and lagging chromosomes (**d**) are shown. Dashed circles highlight the defects in the shSETD1A cells. shGFP cells are shown as controls. The scale bar represents 5 µm. Source data are provided as a Source Data file. **e**–**g:** Bar graphs provide the quantification of each of the events shown in (**b**–**d**). Total number of mitotic cells evaluated under each category across three experimental replicates is provided below. shSETD1A_av_ represents the mean derived following infection with two different shSETD1A constructs. Data from three independent experiments are presented as Mean + SD; *p < 0.05 by two-tailed unpaired Student’s *t* test. Source data are provided as a Source Data file. **h** l-glutamine withdrawal blocks the proliferation of MDA- MB-231 cells. Each data point represents data from three independent experiments presented as Mean + SD; *p < 0.05 by two-tailed unpaired Student’s *t* test. Source data are provided as a Source Data file. **i** Inhibition of proliferation mitigates the senescence phenotype induced by SETD1A-KD. Senescence of SETD1A-KD cells grown in the presence and absence of L-glutamine (and to which glutamine was re-added after 24 hours after SETD1A-KD) was measured using ß–gal staining. Quantification of ß-gal positive senescent cells in shGFP and shSETD1A_av_ cultures. shSETD1A_av_ represents the average from using two different shSETD1A knockdown constructs for each shSETD1A construct. Data from three independent experiments are presented as Mean + SD; *p < 0.05 by two-tailed unpaired Student’s *t* test. Source data are provided as a Source Data file

We then evaluated whether depletion of SETD1A in cells leads to mitotic defects. Dual tubulin and DAPI nuclear staining of SETD1A-KD cells shows a dramatic increase in the fraction of cells harboring micronuclei or chromosomal fragments (Fig. 4a, bar graph shows data from three independent experiments presented as Mean + SD; *p < 0.05 by two-tailed unpaired Student’s *t* test). Real-time imaging of RFP-tagged H2B-expressing cells shows that mitotic defects precede the senescence phenotype exhibited by the SETD1A-KD cells (Supplementary movie 1, 2). Further analysis by co-staining with DAPI and antibodies against CREST, a protein marking the centromeres, also shows pronounced abnormalities in chromosome alignment during metaphase (Fig. 4b, c, e, f; bar graphs show data from three independent experiments presented as Mean + SD; *p < 0.05 by two-tailed unpaired Student’s *t* test). These are measured as metaphase plate abnormalities (Fig. 4b, e; Arbitrary units; shGFP (*n* = 91 cells): 76 ± 17, shSETD1A_av_ from constructs #1 and #2 (*n* = 91 cells): 98 ± 24; bar graph shows data from three independent experiments presented as Mean + SD; *p < 0.001 by two-tailed unpaired Student’s *t* test) and as the fraction of cells with mis-aligned chromosomes (Fig. 4c, f; shGFP (*n* = 47 cells): 24% ± 3%, shSETD1A_av_ from constructs #1 and #2 (*n* = 89 cells): 41% ± 4%; bar graph shows data from three independent experiments presented as Mean + SD; *p < 0.001 by two-tailed unpaired Student’s *t* test). Evaluation of anaphase cells demonstrates an increased fraction of cells with lagging chromosomes (Fig. 4d, g; Mean + SD; shGFP (*n* = 43 cells): 24% ± 3%, shSETD1A_av_ from constructs #1 and #2 (*n* = 68 cells): 53% ± 7%; bar graphs show data from three independent experiments presented as Mean + SD; *p < 0.01 by two-tailed unpaired Student’s *t* test).

To expand on the observation that mitotic defects in proliferating SETD1A-KD cells are likely to be linked to the induction of the senescent phenotype, we took advantage of the tight dependence of MDA-MB-231 cell proliferation on glutamine supplementation in the culture medium, and their profound growth arrest following its withdrawal. Indeed, L-glutamine withdrawal from MDA-MB-231 cells, which suppresses proliferation (Fig. 4h, Each data point represents data from three independent experiments presented as Mean + SD; *p < 0.05 by two-tailed unpaired Student’s *t* test), mitigates the senescence phenotype observed following SETD1A-KD. Restoring L-glutamine to starved cells 24 hours after shSETD1A transfection triggers proliferation as well as the emergence of senescent cells (Fig. 4i, Data from three independent experiments are presented as Mean + SD; *p < 0.05 by two-tailed unpaired Student’s *t* test). Thus, reduction of SETD1A expression in actively proliferating cells results in severe mitotic defects followed by cellular senescence.

### SKP2 contributes to senescence in SETD1A-KD cells

**Fig. 5:**
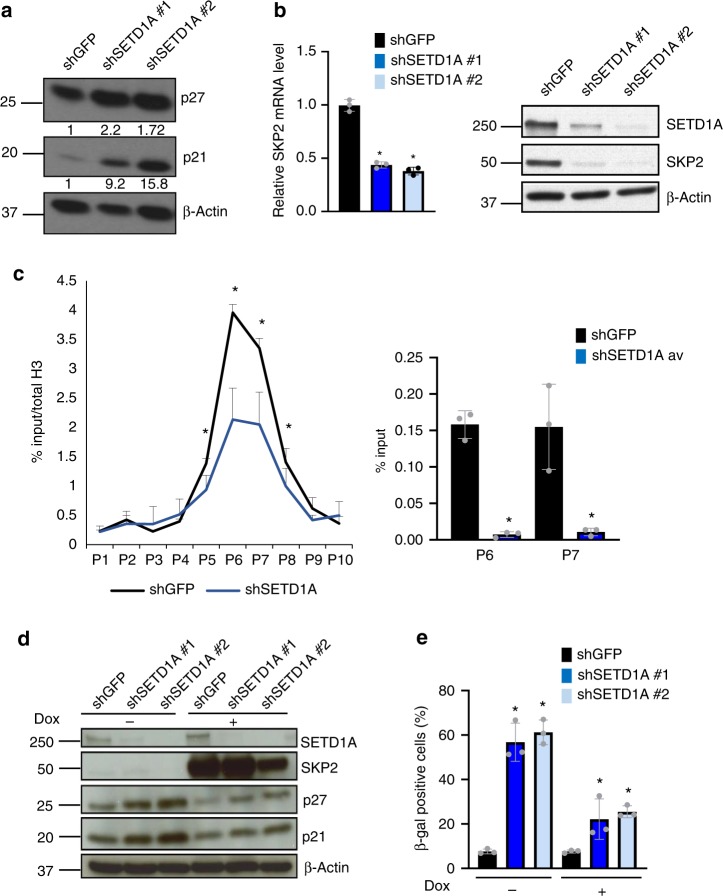
*SKP2* mediates senescence-associated cell cycle arrest in SETD1A-KD cells. **a** Western blot analysis of shGFP- and SETD1A-KDcells shows induction of p21 and p27. ß-actin is shown as control. Quantification of the bands is provided below each blot. **b** SETD1A-KD suppresses SKP2 expression. Left: Bar graph shows the quantification of SKP2 mRNA in shGFP control and shSETD1A cells. Data from three independent experiments are presented as Mean + SD; *p < 0.05 by two-tailed unpaired Student’s *t* test. Source data are provided as a Source Data file. Right: Western blot analysis of SETD1A and SKP2 proteins in shGFP control and SETD1A-KD cells. ß-actin is shown as control. Source data are provided as a Source Data file. **c** Left panel: H3K4Me3 marks on the promoter region of *SKP2* were analyzed using 10 primers (P1–P10) spanning the region in both control (shGFP) and SETD1A-KD cells. The results show that SETD1A-KD suppresses the H3K4Me3 marks on the *SKP2* promoter. Right: Bar graph shows the quantification of SETD1A binding in the promoter regions evaluated with primers P6 and P7. shSETD1A data points represent the average derived from ChIP assays performed with cells individually infected with two different shSETD1A constructs. Data are represented as mean ± SD of the average of three experimental replicates. *p < 0.05 by Mann–Whitney *U* test. Source data are provided as a Source Data file. **d** Overexpression of SKP2 in SETD1A-KD cells suppresses the induction of p27 and p21. SETD1A expression was knocked down in cells following doxycycline-induced expression of SKP2 (Dox + ). The expression of p21 and p27 proteins in cells with and without SKP2 induction and in the presence and absence of SETD1A-KD is shown. ß-actin is shown as loading control. Source data are provided as a Source Data file. **e** Overexpression of SKP2 rescues the senescence phenotype. SETD1A expression was knocked down in cells following the induction of SKP2 expression (Dox + ) and the ß-Gal-positive cells were enumerated. Bar graph shows the percentage of ß-gal positive cells in uninduced and SKP2-induced cells following SETD1A-KD. Data from three independent experiments are presented as Mean + SD; *p < 0.05 by two-tailed unpaired Student’s *t* test. Source data are provided as a Source Data file

Senescence-associated cell cycle arrest in SETD1A-KD cells, despite being independent of the mutational status of *RB* and *p53* (Fig. 2c, Supplementary Fig. 2b), is associated with the induction of p21 and p27 (Fig. 5a). The induction of p21 and p27 protein expression upon SETD1A-KD is also observed in the *RB* and *p53* inactive MBA-MB-468 and BT549 cells (Fig. 2c, Supplementary Fig. 5). Having identified *SKP2*, the ubiquitin ligase which regulates the turnover of these two cell cycle regulators^32^, as a major direct target of SETD1A (Supplementary Fig. 3c), we tested whether SKP2 contributes to the induction of senescence-associated cell cycle arrest in SETD1A-KD cells. Using qPCR and western blot analysis, we first confirmed that endogenous SKP2 mRNA and protein are indeed suppressed in MDA-MB-231 cells following SETD1A-KD (Fig. 5b, Data from three independent experiments are presented as Mean + SD; *p < 0.05 by two-tailed unpaired Student’s *t* test). and then used tiled ChIP-qPCR across the *SKP2* transcriptional start site, identifying the suppression of H3K4Me3 marks in the proximity of the transcription start site (Fig. 5c, Data from three independent experiments are presented as Mean + SD; *p < 0.05 by two-tailed unpaired Student’s *t* test). To determine whether restoration of SKP2 expression in SETD1A-KD cells can rescue the senescence phenotype, we generated cells with ‘doxycycline-inducible SKP2’ expression (Fig. 5d). Inducible expression of SKP2 in SETD1A-KD cells (using two different shSET1A constructs) effectively reduced the expression of p27 and p21 proteins (Fig. 5d). Staining for ß-Galactosidase shows that induction of SKP2 partially suppressed the emergence of ß-Gal-positive senescent cells by 50% following SETD1A-KD (Fig. 5e, Data from three independent experiments are presented as Mean + SD; *p < 0.05 by two-tailed unpaired Student’s *t* test).

### Cells escaping senescence exhibit genomic instability

**Fig. 6:**
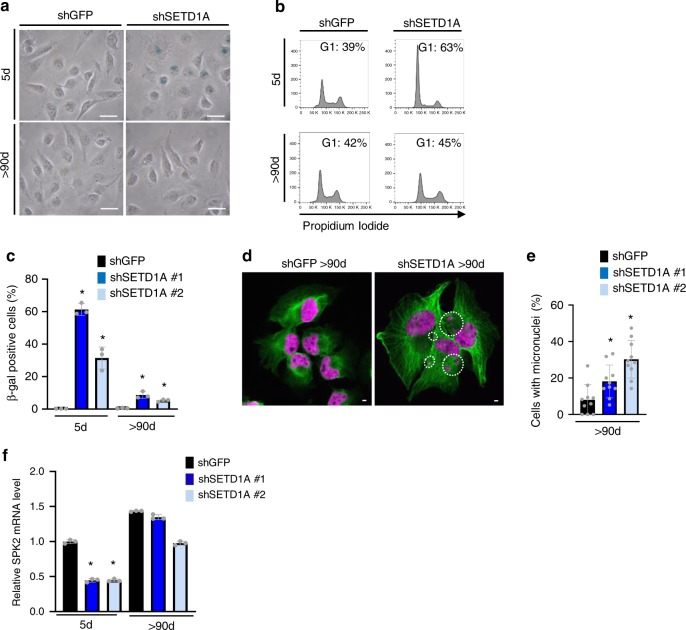
SETD1A-KD cells escape senescence after prolonged culture. **a** SETD1A-KD cells maintained in culture for over 90 days show reduction in the fraction of ß-gal positive cells. Images of ß-gal stained control (shGFP) and SETD1A-KD MDA-MB-231 cells that are senescent (5 days after SETD1A-KD) or escape senescence after prolonged culture ( > 90 days) are shown. The scale bar represents 50 µm. Source data are provided as a Source Data file. **b** Cell cycle analysis of SETD1A-KD cells after 5 days (senescence) or after being maintained for > 90 days in culture (senescence-escape). Escape cells show re-entry into the cell cycle compared with the G1 cell cycle arrest exhibited by SETD1A-KD senescent cells. The quantification of cells in each stage of the cell cycle in the SETD1A-KD senescent and escape cultures is shown in Supplementary Fig. 6b. **c** Bar graph shows the percentage of ß–gal positive cells (Mean + SD) following 5 days (senescence) or > 90 days (Escape) of SETD1A-KD. shGFP-tranduced cells are shown as control for both conditions. Data from two independent experiments are presented as Mean + SD; *p < 0.05 by two-tailed unpaired Student’s t test. Source data are provided as a Source Data file. **d** Confocal images of cells stained with tubulin (green) and DAPI (magenta) show that SETD1A-KD cells escaping senescence harbor chromosome segregation defects visualized as micronuclei (circled). The scale bar represents 50 µm. Source data are provided as a Source Data file. **e** Quantification of SETD1A-KD cells with micronuclei (Mean + SD) under the senescence-escape condition is shown below. shGFP is shown for control. Data from ten different fields per sample are presented as Mean + SD; *p < 0.05 by two-tailed unpaired Student’s *t* test. Source data are provided as a Source Data file. **f** SKP2 mRNA expression is restored in SETD1A-KD cells escaping senescence. Bar graph shows quantification of SKP2 mRNA in cells following 5 days (senescence) and 90 days (Escape) of SETD1A-KD. SKP2 expression in shGFP-transduced cells is shown as control. Data from three independent experiments are presented as Mean + SD; *p < 0.05 by two-tailed unpaired Student’s t test. Source data are provided as a Source Data file

The link between SETD1A and SKP2 in mediating cellular senescence is further supported by the analysis of rare SETD1A-depleted cells that escape senescence after prolonged culture for over 90 days. The SETD1A-KD cells that escape senescence re-enter the cell cycle and resume proliferation and have reduced levels of ß-gal staining (Fig. 6a–c; Supplementary Fig. 6a, 6b, Data from three independent experiments are presented as Mean + SD; *p < 0.05 by two-tailed unpaired Student’s *t* test). Since SETD1A-KD cells show extensive mitotic defects, we tested whether cells escaping senescence would exhibit genomic instability. Staining these cells with tubulin and DAPI demonstrated that these cycling cells harbor major chromosome segregation defects (Fig. 6d, e, Data collected from 10 different fields per sample are presented as Mean + SD; *p < 0.05 by two-tailed unpaired Student’s *t* test). We confirmed that the escaping cells had initially suppressed SETD1A by performing single-cell cloning of control shGFP and SETD1A-KD cells and monitoring colony growth. Our results show that only 2 out of 190 (1.1%) SETD1A-KD cells gave rise to colonies compared with 89.4% in shGFP treated single cells (Supplementary Fig. 6c), suggesting that escape from senescence is a rare event in the model. qPCR analysis confirmed the continued presence of the shSETD1A construct within the cells escaping senescence (Supplementary Fig. 6d). In these senescence-escaping cells SETD1A expression remains only partially suppressed (Supplementary Fig. 6e, Data from three independent experiments are presented as Mean + SD; *p < 0.05 by two-tailed unpaired Student’s *t* test), and SKP2 mRNA expression is restored (Fig. 6f, Data from three independent experiments are presented as Mean + SD; *p < 0.05 by two-tailed unpaired Student’s *t* test). To further determine whether recalibration of SETD1A and SKP2 expression in the SETD1A-KD senescent cells contributes to their escape, we knocked down their expression individually. In both instances, knockdown of either SETD1A or SKP2 re-induces the senescence phenotype (Supplementary Fig 6f, 6g) suggesting that the SETD1A-SKP2 axis maintains its important role in protecting cells from senescence. Unlike the SETD1A-KD senescent cells in which p27 expression is increased, the p27 protein levels in the senescence-escaping SETD1A-KD cells ( > 90 days) are similar to the control shGFP cells (Supplementary Fig. 7). Taken together, these data indicate that rare cells may overcome SETD1A-KD-mediated senescence by reactivating SKP2 expression, partially through recalibrating SETD1A expression, but that they sustain persistent genomic instability, consistent with deregulation of the normal mitotic program.

## Discussion

**Fig. 7:**
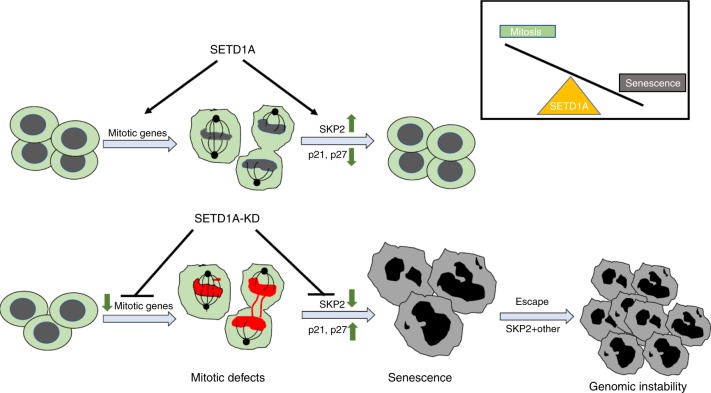
SETD1A maintains the balance between proliferation and senescence. Suppression of SETD1A leads to mitotic defects and simultaneous repression of SKP2 in these cells causes the defective daughter cells to enter senescence with increased levels of p21 and p27. SETD1A-KD cells escaping senescence re-enter the cell cycle through upregulation of SKP2 as well as other mechanisms. The inset summarizes the pivotal role SETD1A expression in maintaining the balance between mitosis and senescence

In summary, we report that knockdown of a single H3K4 methyltransferase, SETD1A, is sufficient to induce massive senescence in proliferating cells. We propose that this phenomenon stems from two convergent pathways: aberrant expression of genes critical for proper execution of mitosis, together with overexpression of the ubiquitin ligase SKP2, whose targets p21 and p27 are known mediators of cell cycle arrest as well as senescence^32^ (Fig. 7). These signals trigger senescence only in proliferating cells, given the absence of this phenotype in growth arrested cells. Taken together, these observations suggest that *SETD1A* plays a critical role in integrating the diverse components of cell division. Such a model would be consistent with *SETD1A* being required for embryonic, epiblast and neural stem cell survival^20^ and the profound proliferative defects that accompany its loss in model organisms^15–19^.

Members of the COMPASS family are the most frequently mutated or rearranged chromatin regulators in many cancers^33^. Prevalence of mutations or translocations of *SETD1A* have not yet been reported cancer, however, *SETD1A* is amplified in mixed ductal and lobular and invasive breast carcinomas, suggesting that increased copy number might constitute one of the mechanisms responsible for its overexpression in cancer. Whether deregulation of DNA methylation could also elevate SETD1A expression in proliferating cancer cells remains to be determined. Cancer cells exhibit continued proliferation, often under conditions of mitotic stress and DNA damage. In this context, overexpression or even amplification of *SETD1A* may serve to confer enhanced fitness, allowing cells to tolerate some deregulation of mitotic pathways. In fact, amplification of the *SETD1A* gene in a high fraction (24%) of patient-derived breast cancer clones engrafting in mice could be attributed to the mitotic and cell cycle competence acquired by these high SETD1A expressing tumor cells^21^.

Although senescence-associated cell cycle arrest is mostly irreversible, a minor fraction of cells does escape senescence and these cells often exhibit more aggressive phenotypes^12,34^. Identifying mechanisms that promote the escape from senescence and re-entry of cells into the cell cycle is critical to maximally exploit the tumor suppressive properties of senescence. Progression of premalignant lesions into malignant tumors predominantly occurs through the loss of *p53*, *INK4a* and *ARF*^35^, whereas mechanisms enabling therapy-induced senescent cells to escape cell cycle arrest are highly context dependent and include the activation of CDK1/cdc2^36^, maintenance of stem cells through ATR and/or Wnt-dependent^37,38^ pathways as well as activation of survival mechanisms^39^. In the SETD1A-KD senescence model, we identify restoration of SKP2 as one of the mechanisms that facilitates the escape of a rare population of mitotically defective cells. Recalibration of SETD1A expression may contribute to restoration of SKP2 expression in the senescence-escaping cells, since repeat knockdown either SETD1A or SKP2 can re-establish the senescence phenotype. We had previously shown that SETD1A, through the induction of a network of miRNAs, suppresses the expression of several p53 downstream targets including BTG2^13^, an inhibitor of cell cycle progression^40^. Knockdown of BTG2 in SETD1A-KD cells also decreases the fraction of ß-gal positive cells implicating additional mechanisms in the escape of SETD1A-KD cells from senescence (Supplementary Fig. 8, Data from three independent experiments are presented as Mean + SD; *p < 0.05 by two-tailed unpaired Student’s *t* test)

Senescence is not only a potent tumor-suppressive and drug-response mechanism, it is also thought to be a fundamental cellular pathway that drives organismal aging^3,4^. As such, *SETD1A*, a chromatin modifying enzyme which integrates two fundamentally opposing pathways – proliferation and senescence – provides a druggable node that may provide insight into modulating cancer and aging phenotypes in the future. Histone-modifying enzymes, which regulate transcriptional programs, are emerging as promising cancer therapeutic targets^41–43^. Our findings show that inhibiting SETD1A is sufficient to unleash the senescence phenotype illustrating the potential consequence of targeting a single chromatin modifier. For cancer applications, exploiting the senescence phenotype induced by SETD1A inhibition will require identifying additional actionable signaling nodes to suppress escape from senescence, and combinatorial regimens to definitively halt cancer cell proliferation.

## Methods

### Cell culture

All cell lines were acquired from ATCC. Human breast and lung cancer cell lines, MDA-MB231, A549, MCF7, MDA-MB-468, BT549, and H1299, were grown in Dulbeccco’s modified medium supplemented with 10% female fetal bovine serum, glutamine and penicillin/streptomycin. Human colon cancer cell lines, HCT8, H630, HCT116, DLD1, HCT15, SW620 and HT29, were grown in RPMI 1640 medium supplemented with 10% female fetal bovine serum and penicillin/streptomycin. They were periodically authenticated and were matched with the earliest passage cell lines. All cell lines were also periodically tested for mycoplasma contamination using the MycoFluorTM Mycoplasma Detection Kit (Thermo Fisher Scientific) and shown as negative for infection.

### Viral infection

Lentiviral short hairpin RNA constructs were obtained from the RNAi Consortium shRNA Library at the Broad Institute. The target sequences against each gene are listed in the Table 1. Inducible expression of SKP2 was accomplished by stable lentiviral transfection of pInducer-SKP2 into cells. The sequence pENTR-*SKP2*, Ultimate ORF from Life Technologies, was transferred to the backbone pInducer 20 with the gateway system from Life Technologies. Conditioned medium containing infective lentiviral particles was generated by co-transfecting 3 µg of lentiviral vector, 3 µg of pCMV ∂8.91 and 1 µg pHCMV-VSV-G into 2 × 10^6^ 293 T human embryonic kidney cells using FuGENE 6 transfection reagent (Roche Applied Science). Supernatants were collected 48 hours after transfection and filtered through a 0.45 um membrane (Millipore). The cells were directly infected using 8 µg/mL of polybrene.

**Table 1:** List of target sequences against shRNAs

	5′–3′
SETD1A #1	CCGGGAAGATCGTGATCTACTCCAACTCGAGTTGGAGTAGATCACGATCTTCTTTTTTG
SETD1A #2	CCGGGCGATTCGTCTTCCAAATGTTCTCGAGAACATTTGGAAGACGAATCGCTTTTTTG
SKP2 #1	CCGGGCCTAAGCTAAATCGAGAGAACTCGAGTTCTCTCGATTTAGCTTAGGCTTTTTG
SKP2 #2	CCGGGATAGTGTCATGCTAAAGAATCTCGAGATTCTTTAGCATGACACTATCTTTTTG

### RNA interference assay

siRNA against BTG2 (5′-CAGAGCACUACAAACACCACUGGUU-3′) or non-targeting control (50 nM) were transfected into cells using the Lipofectamine RNAiMAX (Invitrogen) according to manufacturer’s protocol.

### qPCR

RNA was isolated using RNeasy Mini kit (Qiagen) and mRNA quantitation was performed using SYBR Green in an ABI PRISM 7500 sequence detection system with 96-block module and automation accessory (Applied Bio-system). GAPDH or ß-actin was used as an internal control. All samples were analyzed in triplicate. The primer sequences are listed in the Table 2.

**Table 2:** The primer sequences used for gene expression analysis using qPCR

SETD1A	Forward	5′-GGCCAGATTCATCAACCACT-3′
SETD1A	Reverse	5′-CGATCTTCTTCTGGGACTCG-3′
SKP2	Forward	5′-ATGCCCCAATCTTGTCCATCT-3′
SKP2	Reverse	5′-CACCGACTGAGTGATAGGTGT-3′
GAPDH	Forward	5′-AGTCCTTCCACGATACCAAAGT-3′
GAPDH	Reverse	5′-CATGAGAAGTATGACAACAGCCT-3′
β-Actin	Forward	5′-CTCTTCCAGCCTTCCTTCCT-3′
β-Actin	Reverse	5′-AGCACTGTGTTGGCGTACAG-3′
BTG2	Forward	5′-CAGAGCACTACAAACACCACTG-3′
BTG2	Reverse	5′-CTGAGTCCGATCTGGCTGG-3′

### Western blot

Antibodies used were rabbit anti-SETD1A polyclonal antibody (Cat. A300–289 A, BETHYL; 1:1000 dilution), rabbit anti-p21 polyclonal antibody (Cat. sc-397, Santa Cruz; 1:100 dilution), rabbit anti-p27 polyclonal antibody (Cat. sc-528, Santa Cruz; 1:100 dilution), rabbit anti-caspase-3 polyclonal antibody (Cat. sc-7148; 1:200 dilution), rabbit anti-PARP antibody (Cat. #9542; Cell Signaling Technology; 1:1000 dilution), rabbit anti-Histone H3 antibody (Cat. ab176842, Abcam; 1:500 dilution), rabbit anti-SKP2 antibody (Cat. #4358; Cell Signaling), rabbit anti-Histone H3 mono-methyl K4 antibody (Cat. ab8895; Abcam; 1:500 dilution), rabbit anti-Histone H3 di-methyl K4 antibody (Cat. ab32356; Abcam; 1:2000 dilution), rabbit anti-Histone H3 tri-methyl K4 antibody (Cat. ab12209; Abcam; 1:1000 dilution) and mouse anti-Actin monoclonal antibody (BD Bioscience; 1:1000 dilution). Cell were lysed in RIPA lysis buffer [20 mM Tris, pH8.0 150 mM NaCl, 10 mM NaF, 0.1% SDS, 1% Nonidet P-40, 1X protease inhibitor mixture (Roche)]. Lysates were run on a SDS-4-15% polyacrylamide gel (Bio-Rad) and transferred onto PVDF membranes (Millipore) and Nitrocellulose membrane (Invitrogen). Immunoblots were visualized with a Western Lightning Plus chemiluminescence kit (PerkinElmer) and the Odyssey blot imager (LI-COR). Uncropped and unprocessed scans of all the important blots are provided in the source data file.

### Senescence-associated (SA) ß-galactosidase staining

SA ß-galactosidase staining was performed using the Senescence Cells Histochemical Staining Kit from Sigma-Aldrich. Briefly, cells were fixed and incubated with freshly prepared staining solution overnight according to manufacturer instructions. The percentage of positively stained cells was determined by counting five random fields. Images of representative fields were captured under 200X magnification.

### Cell cycle analysis

Cells were harvested, washed twice in PBS, resuspended in 70% ethanol overnight, and then diluted in propidium iodide, RNase staining buffer (BD Pharmingen) and incubated for 15 min at 37 °C. Samples were analyzed with flow cytometry (BD LSRFortessa Cell analyzer) and Flowjo software (FLOWJO, LLC).

### Time-lapse imaging

H2B-RFP-expressing A549 cells were infected with either shSETD1A or shGFP and real-time imaging was initiated 24 hours after infection using the confocal Zeiss LSM170 using an enclosed stage incubator, after regulating temperature, CO_2_ level and humidity. For RFP images within each well, nine images (3 × 3 tile) were captured every 5–8 min over 3 days (24–96 h after infection) at ×20 magnification. Sample focus was maintained during the course of the experiment using an external diode laser. Cell viability was confirmed by observing of mitotic cells throughout the duration of the experiment. Videos were analyzed using the ZEN software (Zeiss).

### Proliferation assays

Cells were trypsinized and 2.5 × 10^3^ cells were plated in individual wells of a 96-well plate. On the day of evaluation, a 10 µL WST-8 solution (WST-8 cell proliferation and cytotoxicity assay kit; Cayman Chemical) was added into each well. Plates were then incubated for an additional 1 h at 37 °C and the absorbance was determined at 450 nm. The cell counts for 3 wells/time-point were averaged for each group and the data were used to derive growth curves.

### ChIP-Sequencing

ChIP assay to define H3K4Me3 marks on the SKP2 promoter using 10 primers spanning the region was done as described in^13^. Quantitative PCR was performed using primers listed in the Table 3.

**Table 3:** DNA sequences of the 10 primers spanning the *SKP2* promoter

P1	Forward	5′-TTCCAAGCACCATTCATTCA-3′
	Reverse	5′-GTGGTGGCAGCTACCTGTTT-3′
P2	Forward	5′-AAGAAGGGTGGACCGTCTTC-3′
	Reverse	5′-GTCAGGCTGGTCTCGAACTC-3′
P3	Forward	5′-GTTACCGGGGCAAACTATCA-3′
	Reverse	5′-CTGGGAACTAGAATACTTGCAACA-3′
P4	Forward	5′-GGGAAAGGACGTAGCTTCAA-3′
	Reverse	5′-AGGCTAAGCCGTTCATCAAA-3′
P5	Forward	5′-ATGGATTTCGCATGGTCATT-3′
	Reverse	5′-TCAGCTGGCTACGTGTGTTT-3′
P6	Forward	5′-GCTGGGCTACTGTCACCACT-3′
	Reverse	5′-GTGAGGCGCTTTTGAGTCTG-3′
P7	Forward	5′-TGCTAGGCTTAGCGGGTCT-3′
	Reverse	5′-CCCTTTTTGCAATCCGTTTA-3′
P8	Forward	5′-TGCGATTCTGTTAGCTGCTG-3′
	Reverse	5′-CTTCCTGCAGAAGTGCACAA-3′
P9	Forward	5′-GGGCAAGTCGTCAAGTATGC-3′
	Reverse	5′-GCAGGCAGATTCCCTTCTAA-3′
P10	Forward	5′-TTAATCACCCAACCCGAAAA-3′
	Reverse	5′-GGGATAGACCTGGGGAGAAG-3′

Distribution of global histone H3 harboring H3K4me3 marks in shGFP and SETD1A-KD cells (from three independent samples) was determined by ChIP-seq^44^. Cells at 80% confluence in 150 mm dishes (3 × 10^6^- 5 × 10^6^ cells per dish) were crosslinked with 1% formaldehyde for 15 min at 37 °C and quenched with formaldehyde containing 125 mM glycine. The cells were then washed twice with cold PBS and collected into 1 mL of cellular lysis buffer (5 mM Pipes, 85 mM KCl, and 0.5% NP-40 with protease inhibitors mixture). Nuclei were collected and incubated in nuclear lysis buffer [50 mM Tris (pH 8.0), 10 mM EDTA (pH 8.0), 0.2% EDTA with protease inhibitors mixture]. Formaldehyde-crosslinked chromatin was fragmented to a target size of 200-300 bp using a Covaris S2 sonicator (Covaris, Woburn, MA) at 4 °C. Size distribution of gDNA fragments was determined using the Agilent Bioanalyzer (Agilent Technologies, Santa Clara, CA). Protein G magnetic beads were incubated with 4 µg of either anti-Histone H3 tri-methyl K4 antibody or IgG control on rotator at 4 °C for 6 h. Sonicated chromatin was subjected to chromatin immunoprecipitation with protein G magnetic beads conjugated with each antibody on rotator at 4 °C overnight. After six washes, the beads were eluted with elution buffer (50 mM NaHCO_3_, 140 mM NaCl, 1% SDS). Following both RNaseA and proteinase K treatment, genomic DNA fragments enriched for interaction with H3K4me3 were released from proteins by heat, purified using PCR clean-up kit (Qiagen), and subjected to deep sequencing library construction using the SOLiD 5500 Fragment Library Core Kit and the SOLiD EZ Bead emulsion PCR system (Life Technologies, Carlsbad, CA). Deep sequencing was performed using the SOLiD 5500XL deep sequencer (50 nt, single reads), and the XSQ-format raw data were subjected to color-space alignment to the GRCh37/hg19 human genome reference sequence to obtain the BAM format-aligned read data using the ABI LifeScope software (Life Technologies). Uniquely mapped reads (12-15 million reads per library) were subjected to detection of H3K4me3 peaks and differential distributions using the MACS2 *callpeak* function^45^. Annotation of H3K4me3 peaks to the transcription start sites of known human genes was performed using the R/bioconductor packages *ChIPseeker*^46^, *clusterProfiler*^47^, and *TxDb.Hsapiens.UCSC.hg19.knownGenes*. Gene Ontology analysis of genes differentially enriched for H3K4me3 at the promoter sequence was performed using the DAVID web server^48^. Quantitative differences in promoter-associated H3K4me3 peaks within known genes were statistically examined using two-samples *t* test with Welch’s correction. ChIP sequencing data have been deposited in the NCBI Gene Expression Omnibus database under accession code GSE 117427.

### Arrayed gene expression analysis

RNA expression in SETD1A-depleted A549 and MDA-MB-231 cells was analyzed using the Human Gene Expression 12 × 135 K Arrays (Roche Nimblegen). Briefly RNA was extracted from A549 and MDA-MB-231 cells infected with shGFP or two independent shSETD1A constructs (shSETD1A#1 and shSETD1A#2) using the RNeasy Mini kit (Qiagen). cDNA synthesis was performed using the Roche cDNA synthesis system (11 117 831 001). cDNA was hybridized to the Human Gene Expression 12 × 135 K Arrays in duplicate according to the manufacturer’s protocol. The microarrays were scanned on Nimblegen MS200 at 2 µm resolution. Scans were converted to RMA-normalized^49,50^ expression values using Roche NimbleScan 2.6 software. Microarray data have been deposited in the NCBI Gene Expression Omnibus database under accession code GSE 71498^13^.

### Quantitative proteomics

To define the mechanism underlying the senescence phenotype exhibited by SETD1A knockdown cells, we quantitatively mapped the proteomes using multiplexed mass spectrometry (MS) by applying isobaric tandem mass tag (TMT) technology^26,27^. shGFP and shSETD1A MDA-MB-231 and A549 cells (carried out in triplicate) were pelleted and re-suspended in lysis buffer containing 75 mM NaCl, 50 mM HEPES (pH 8.5), 10 mM sodium pyrophosphate, 10 mM NaF, 10 mM ß-glycerophosphate, 10 mM sodium orthovanadate, 10 mM phenylmethanesulfonylfluoride, Roche Complete Protease Inhibitor EDTA-free tablets, and 3% sodium dodecyl sulfate. Cells were lysed by passing them 10 times through a 21-gauge needle, and the lysates were prepared for analysis on the mass spectrometer^28^. Briefly, reduction and thiol alkylation were followed by purifying the proteins using MeOH/CHCl3 precipitation. Protein digest was performed with Lys- C and trypsin, and peptides were labeled with TMT-10-plex reagents (Thermo Scientific) and fractionated by basic pH reversed phase chromatography. Multiplexed quantitative proteomics was performed on an Orbitrap Fusion mass spectrometer (Thermo Scientific) using a Simultaneous Precursor Selection (SPS) based MS3 method^26,27^. MS2 spectra were assigned using a SEQUEST-based proteomics analysis platform^51^. Based on the target-decoy database search strategy^52^ and employing linear discriminant analysis and posterior error histogram sorting, peptide and protein assignments were filtered to a false discovery rate (FDR) of <1%^53^. Peptides with sequences that were contained in more than one protein sequence from the UniProt database were assigned to the protein with most matching peptides^53^. TMT reporter ion intensities were extracted as that of the most intense ion within a 0.03 Th window around the predicted reporter ion intensities in the collected MS3 spectra. Only MS3 with an average signal- to-noise value larger than 40 per reporter ion as well as with an isolation specificity^27^ larger than 0.75 were considered for quantification. A two-step normalization of the protein TMT-intensities was performed by first normalizing the protein intensities over all acquired TMT channels for each protein based on the median average protein intensity calculated for all proteins. To correct for slight mixing errors of the peptide mixture from each sample, a median of the normalized intensities was calculated from all protein intensities in each TMT channel, and protein intensities were normalized to the median value of these median intensities. Known protein-protein interactions were extracted from the String database (high confidence score > 0.7)^54^.

### Analysis of gene sets enriched in SETD1A co-regulated genes

Data derived from multiplexed proteomic analysis on 41 breast cancer cell lines described in ref. ^28^ were used to calculate the co-regulation of all proteins with respect to endogenous basal SETD1A protein expression using pairwise Pearson correlation calculations^28^. The correlation scores were sorted to generate an ordered list of proteins and then the proteins identifiers were converted to their associated gene symbol. We performed a GSEA for the GO term Biological Process gene set collection (MSigDB) using the pre-Ranked GSEA tool available from the Broad^55^. Significantly enriched genesets were defined by the Benjamini-Hochberg adjusted *p* values (FDR *q* values < 25%). The full set of significantly enriched gene sets are provided as Supplementary table 1.

### SETD1A co-dependency analysis

The Project Achilles RNAi viability database was analyzed to identify the gene dependencies that are most closely associated with SETD1A dependency. This analysis was performed on the published Achilles v2.20.2 dataset, comprised of 17,098 gene dependency z-score across 501 cancer cell lines; however, since SETD1A dependency was only measured in a subset of 285 cell lines, only this subset was analyzed^56^. Gene dependencies were ranked by Pearson correlation coefficient with SETD1A dependency across 285 lines, and false discovery rate (FDR) was controlled by the Benjamini and Hochberg method. The overlap of positively correlated gene dependencies (FDR < 0.01) with GO gene sets was evaluated using Gene Set Enrichment Analysis (GSEA, MSigDB v6.1, c5 gene sets)^55,57^. For gene dependencies represented in multiple enriched and related GO gene sets, a heatmap was generated to depict the association with SETD1A dependency, comparing the gene dependency z-scores in cell lines that are SETD1A-dependent (*z*-score < -2) and SETD1A-independent (*z*-score > + 2).

### Immunofluorescence analysis

Cells were plated overnight on a 4 or 8-well chamber slide (Millipore or LAB-TEK) coated with poly-L-lysine (Sigma), fixed with 4% paraformaldehyde and washed with PBS. Fixed cells were then permeabilized with 0.5% Triton X-100 in PBS, blocked with 5% BSA, and stained with mouse anti-Tubulin antibody (NOVUS; 1:200 dilution) or human anti-Centromere antibody (Antibody Inc; 1:200 dilution) for 1 h, and incubated with secondary antibodies conjugated with Alexa Fluor dyes (1:500 dilution) for 1 h. Coverslips were then mounted with anti-fade agent Prolong with 4′,6-diamidino-2-phenylindole (DAPI) (Invitrogen). Photomicrographs were taken with the NIKON 90i microscope or with the confocal Zeiss LSM170. Analysis and quantification of the mitotic events of interest were performed with ImageJ software (NIH).

### Statistics

The statistical analyses performed is described in the figure legends. The differences between the means were considered to be statistically significant at *P* < 0.05.

### Reporting summary

Further information on research design is available in the Nature Research Reporting Summary linked to this article.

## Supplementary information

Supplementary_info_NEWv2

Reporting Summary

Description of Additional Supplementary Files

A549 shControl

A549 shSETD1A

Source Data

## Data Availability

Microarray data has been deposited in the NCBI Gene Expression Omnibus database under accession code GSE71498. The proteomics data have been deposited to the ProteomeXchange Consortium via the MassIVE partner repository with the dataset identifier MSV000083763. The source data underlying Figs. 1a, 1c, d, 2a–c, 3d, 4a–i, 5a–e, 6a, 6c–g and Supplementary Figs. 1a–d, 2a, 3c, 5, 6a–c, 6e–gd, 7 and 8 are provided as a Source Data file. All the other data supporting the findings of this study are available within the article and its Supplementary Information files and from the corresponding author upon reasonable request.

## References

[1] Campisi J, d’Adda di Fagagna F (2007). Cellular senescence: when bad things happen to good cells. Nat. Rev. Mol. Cell Biol..

[2] Kuilman T, Michaloglou C, Mooi WJ, Peeper DS (2010). The essence of senescence. Genes Dev..

[3] Campisi J (2005). Senescent cells, tumor suppression, and organismal aging: good citizens, bad neighbors. Cell.

[4] Jeyapalan JC, Sedivy JM (2008). Cellular senescence and organismal aging. Mech. Ageing Dev..

[5] Choi J (2000). Expression of senescence-associated beta-galactosidase in enlarged prostates from men with benign prostatic hyperplasia. Urology.

[6] Collado M (2005). Tumour biology: senescence in premalignant tumours. Nature.

[7] Majumder PK (2008). A prostatic intraepithelial neoplasia-dependent p27 Kip1 checkpoint induces senescence and inhibits cell proliferation and cancer progression. Cancer Cell.

[8] Michaloglou C (2005). BRAFE600-associated senescence-like cell cycle arrest of human naevi. Nature.

[9] Achuthan S, Santhoshkumar TR, Prabhakar J, Nair SA, Pillai MR (2011). Drug-induced senescence generates chemoresistant stemlike cells with low reactive oxygen species. J. Biol. Chem..

[10] Mosieniak G (2015). Polyploidy formation in doxorubicin-treated cancer cells can favor escape from senescence. Neoplasia.

[11] Ewald JA, Desotelle JA, Wilding G, Jarrard DF (2010). Therapy-induced senescence in cancer. J. Natl Cancer Inst..

[12] Gordon RR, Nelson PS (2012). Cellular senescence and cancer chemotherapy resistance. Drug Resist Updat.

[13] Tajima K (2015). SETD1A modulates cell cycle progression through a miRNA network that regulates p53 target genes. Nat. Commun..

[14] Shilatifard A (2012). The COMPASS family of histone H3K4 methylases: mechanisms of regulation in development and disease pathogenesis. Annu Rev. Biochem.

[15] Ardehali MB (2011). Drosophila Set1 is the major histone H3 lysine 4 trimethyltransferase with role in transcription. EMBO J..

[16] Briggs SD (2001). Histone H3 lysine 4 methylation is mediated by Set1 and required for cell growth and rDNA silencing in Saccharomyces cerevisiae. Genes Dev..

[17] Hallson G (2012). dSet1 is the main H3K4 di- and tri-methyltransferase throughout Drosophila development. Genetics.

[18] Mohan M (2011). The COMPASS family of H3K4 methylases in Drosophila. Mol. Cell Biol..

[19] Nislow C, Ray E, Pillus L (1997). SET1, a yeast member of the trithorax family, functions in transcriptional silencing and diverse cellular processes. Mol. Biol. Cell.

[20] Bledau AS (2014). The H3K4 methyltransferase Setd1a is first required at the epiblast stage, whereas Setd1b becomes essential after gastrulation. Development.

[21] Eirew P (2015). Dynamics of genomic clones in breast cancer patient xenografts at single-cell resolution. Nature.

[22] Hernandez-Segura A (2017). Unmasking Transcriptional Heterogeneity in Senescent Cells. Curr. Biol..

[23] Kaufmann SH, Desnoyers S, Ottaviano Y, Davidson NE, Poirier GG (1993). Specific proteolytic cleavage of poly(ADP-ribose) polymerase: an early marker of chemotherapy-induced apoptosis. Cancer Res..

[24] Nicholson DW (1995). Identification and inhibition of the ICE/CED-3 protease necessary for mammalian apoptosis. Nature.

[25] Childs BG, Durik M, Baker DJ, van Deursen JM (2015). Cellular senescence in aging and age-related disease: from mechanisms to therapy. Nat. Med..

[26] McAlister GC (2014). MultiNotch MS3 enables accurate, sensitive, and multiplexed detection of differential expression across cancer cell line proteomes. Anal. Chem..

[27] Ting L, Rad R, Gygi SP, Haas W (2011). MS3 eliminates ratio distortion in isobaric multiplexed quantitative proteomics. Nat. Methods.

[28] Lapek JD (2017). Detection of dysregulated protein-association networks by high-throughput proteomics predicts cancer vulnerabilities. Nat. Biotechnol..

[29] Aceto N (2014). Circulating tumor cell clusters are oligoclonal precursors of breast cancer metastasis. Cell.

[30] Aceto N (2018). AR expression in breast cancer CTCs associates with bone metastases. Mol. Cancer Res.

[31] Hoshii T (2018). A non-catalytic function of SETD1A regulates cyclin k and the dna damage response. Cell.

[32] Lin HK (2010). Skp2 targeting suppresses tumorigenesis by Arf-p53-independent cellular senescence. Nature.

[33] Ford DJ, Dingwall AK (2015). The cancer COMPASS: navigating the functions of MLL complexes in cancer. Cancer Genet.

[34] Kahlem P, Dorken B, Schmitt CA (2004). Cellular senescence in cancer treatment: friend or foe?. J. Clin. Invest.

[35] Collado M, Serrano M (2010). Senescence in tumours: evidence from mice and humans. Nat. Rev. Cancer.

[36] Roberson RS, Kussick SJ, Vallieres E, Chen SY, Wu DY (2005). Escape from therapy-induced accelerated cellular senescence in p53-null lung cancer cells and in human lung cancers. Cancer Res.

[37] Milanovic M (2018). Senescence-associated reprogramming promotes cancer stemness. Nature.

[38] Sabisz M, Skladanowski A (2009). Cancer stem cells and escape from drug-induced premature senescence in human lung tumor cells: implications for drug resistance and in vitro drug screening models. Cell Cycle.

[39] de Carne Trecesson S (2011). Escape from p21-mediated oncogene-induced senescence leads to cell dedifferentiation and dependence on anti-apoptotic Bcl-xL and MCL1 proteins. J. Biol. Chem..

[40] Winkler GS (2010). The mammalian anti-proliferative BTG/Tob protein family. J. Cell Physiol..

[41] Dawson MA, Kouzarides T (2012). Cancer epigenetics: from mechanism to therapy. Cell.

[42] Keppler BR, Archer TK (2008). Chromatin-modifying enzymes as therapeutic targets–Part 2. Expert Opin. Ther. Targets.

[43] Keppler BR, Archer TK (2008). Chromatin-modifying enzymes as therapeutic targets–Part 1. Expert Opin. Ther. Targets.

[44] Landt SG (2012). ChIP-seq guidelines and practices of the ENCODE and modENCODE consortia. Genome Res.

[45] Zhang Y (2008). Model-based analysis of ChIP-Seq (MACS). Genome Biol..

[46] Yu G, Wang LG, He QY (2015). ChIPseeker: an R/Bioconductor package for ChIP peak annotation, comparison and visualization. Bioinformatics.

[47] Yu G, Wang LG, Han Y, He Q (2012). Y. clusterProfiler: an R package for comparing biological themes among gene clusters. OMICS.

[48] Dennis G (2003). DAVID: database for annotation, visualization, and integrated discovery.. Genome Biol.

[49] Irizarry RA (2003). Summaries of Affymetrix GeneChip probe level data. Nucleic Acids Res.

[50] Irizarry RA (2003). Exploration, normalization, and summaries of high density oligonucleotide array probe level data. Biostatistics.

[51] Eng JK, McCormack AL, Yates JR (1994). An approach to correlate tandem mass spectral data of peptides with amino acid sequences in a protein database. J. Am. Soc. Mass Spectrom..

[52] Elias JE, Gygi SP (2007). Target-decoy search strategy for increased confidence in large-scale protein identifications by mass spectrometry. Nat. Methods.

[53] Huttlin EL (2010). A tissue-specific atlas of mouse protein phosphorylation and expression. Cell.

[54] Szklarczyk D (2011). The STRING database in 2011: functional interaction networks of proteins, globally integrated and scored. Nucleic Acids Res..

[55] Subramanian A (2005). Gene set enrichment analysis: a knowledge-based approach for interpreting genome-wide expression profiles. Proc. Natl Acad. Sci. USA.

[56] Tsherniak A (2017). Defining a cancer dependency map. Cell.

[57] Mootha VK (2003). PGC-1alpha-responsive genes involved in oxidative phosphorylation are coordinately downregulated in human diabetes. Nat. Genet.

