# Supplementary material for: SETD1A protects from senescence through regulation of the mitotic gene expression program
Source: Nat Commun. 2019 Jun 28;10:2854. doi: 10.1038/s41467-019-10786-w (PMC6599037; doi:10.1038/s41467-019-10786-w)
Supplement: Supplementary file 1 — Supplementary_info_NEWv2 [file 41467_2019_10786_MOESM1_ESM.pdf]

Supplementary Information

SETD1A protects from senescence through regulation of the mitotic gene expression program

Tajima et al.

Contents

Supplementary figures, Table and Legends

Supplementary Fig. 1. SETD1A-KD induces senescence.

Supplementary Fig. 2. SETD1A-KD induces senescence independent of *p53* and *RB*.

Supplementary Fig. 3. SETD1A regulates mitosis, cell cycle and DNA damage response genes.

Supplementary Fig. 4. Direct visualization of correlation between dependence on genes that mediate sister chromatid cohesion and SETD1A dependence.

Supplementary Fig. 5. SETD1A-KD in *RB* and *p53* mutant cells induces p21 and p27.

Supplementary Fig. 6. SETD1A-KD cells escape senescence.

Supplementary Fig. 7. p21 and p27 expression in SETD1A-KD senescence escaping cells.

Supplementary Fig. 8. BTG2 contributes to SETD1A-mediated senescence.

Supplementary Table 1: Description of the leading-edge proteins related to the processes shown in Fig. S3B

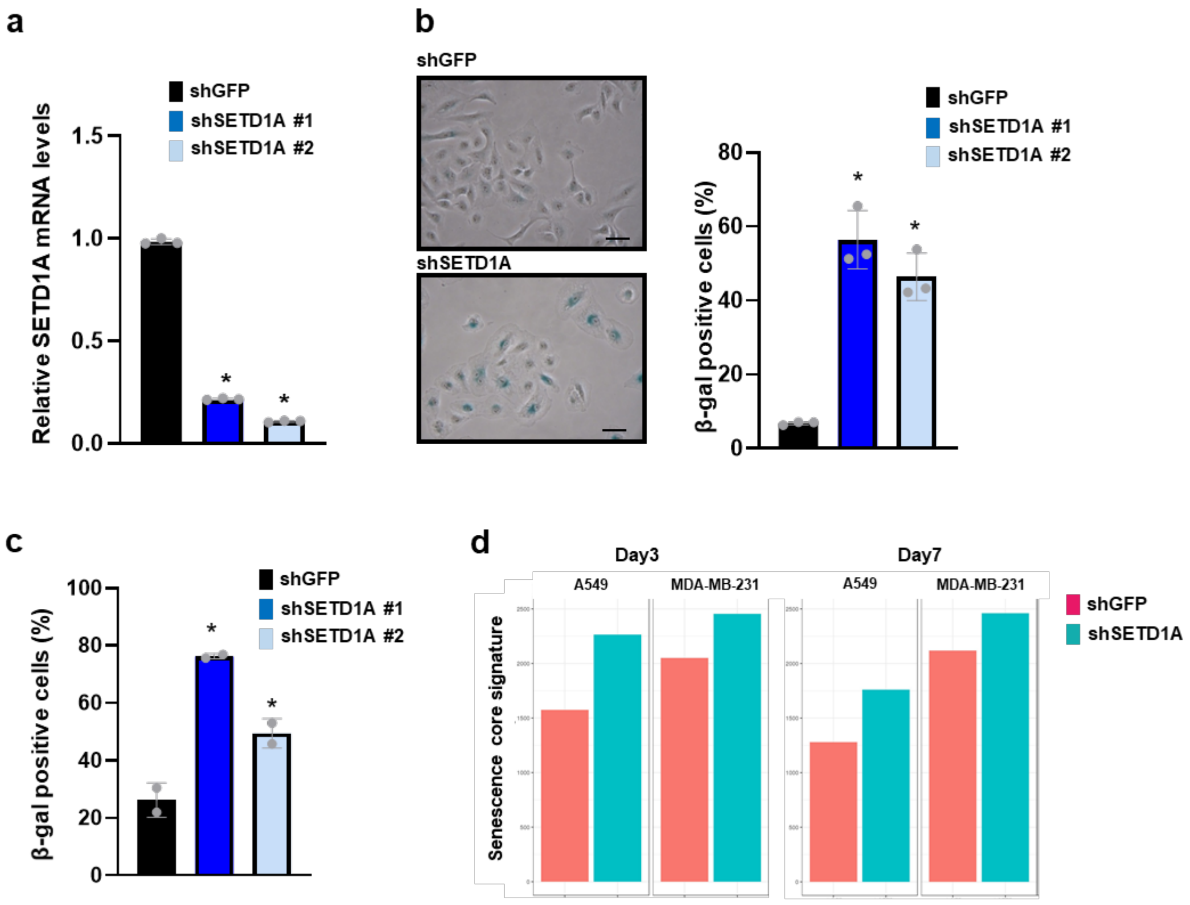

Supplementary Fig. 1. SETD1A-KD induces senescence

1a: Bar graph shows the relative SETD1A mRNA levels in SETD1A-KD MDA-MB-231 cells compared with shGFP-cells. $shSETD1A_{av}$ represents the mean of cells infected with two different shSETD1A constructs. Data from three independent experiments are presented as Mean±SD; *p<0.05 by two-tailed unpaired Student's t test. Source data are provided as a Source data file.

1b: Left: Images of ß-gal stained control (shGFP) and SETD1A-KD (shSETD1A) A549 cells. The scale bar represents 50 µm. Right: Bar graph shows quantification of ß-gal positive cells in the shSETD1A and shGFP cultures. Data from three independent experiments are presented as Mean±SD; *p<0.05 by two-tailed unpaired Student's t test. Source data are provided as a Source data file.

1c: Bar graph shows quantification of ß-gal positive cells (Mean±SD) in the shSETD1A and shGFP expressing immortalized non-tumorigenic MCF10A cells. Data from two independent experiments are presented as Mean±SD; *p<0.05 by two-tailed unpaired Student's t test. Source data are provided as a Source data file.

1d: SETD1A-KD increases the 'senescence core signature'. Expression analysis of the 55 genes comprising the 'senescence core signature' [22] in RNA-Sequencing data derived from A549 and MDA-MB-231 cells after 3 and 7 days or SETD1A-KD. shGFP cells were used as control. Source data are provided as a Source Data file.

a

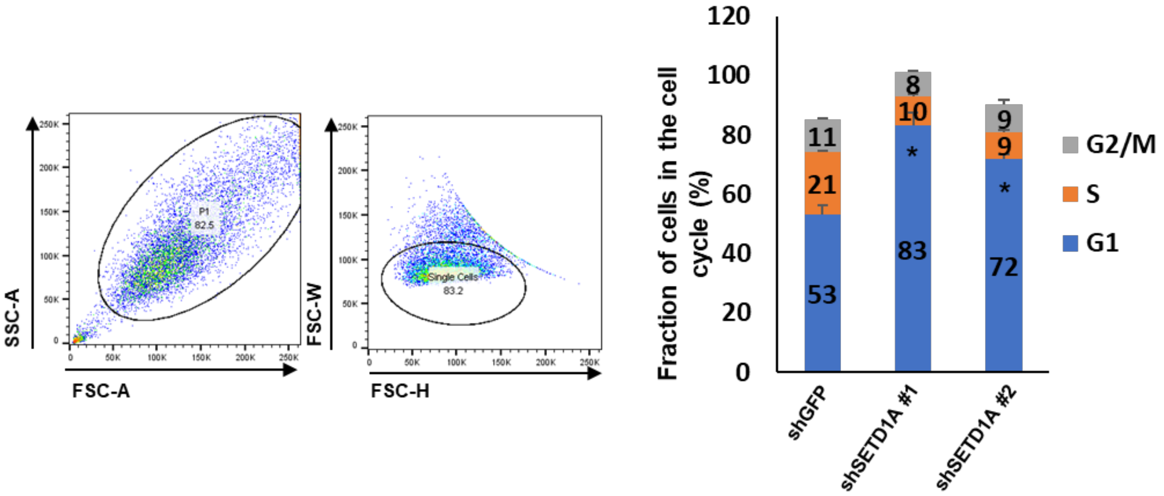

b

cell line	TP53	RB	INK4A	K-Ras	shGFP	shSETD1A av	P value
HCT8	Wt	N/A	wt	Wt	16.34±3.9	25.65±2.65	<0.05
H630	Mut	N/A	wt	wt	1.00±0.32	42.61±6.46	<0.05
HCT116	Wt	wt	insertion	mut	20.72±2.63	49.69±3.31	<0.05
DLD1	Mut	N/A	wt	wt	0.45±0.16	4.51±0.89	<0.05
HCT15	Mut	wt	wt	mut	1.64±0.87	10.29±2.05	<0.05
SW620	Mut	wt	wt	mut	0.43±0.19	4.74±0.73	<0.05
HT29	Mut	wt	wt	wt	0.88±0.34	19.21±4.35	<0.05

Supplementary Fig. 2. SETD1A-KD induces senescence independent of p53 and RB
2a: Depletion of SETD1A induces cell cycle arrest. SETD1A expression was knocked down in MDA-MB-231 cells and the fraction of cells in each phase of the cell cycle was measured by FACS analysis. Left: Graphical account for FACS gating strategies for the figure on the right, which include gating for cell size and single cells. Right: Fraction of cells in G1, S and G2/M are shown. shGFP cells are shown as control. Data from three independent experiments are presented as Mean±SD; *p<0.05 by two-tailed unpaired Student's t test. Source data are provided as a Source data file.
2b: Quantification of ß-gal positive cells in multiple colon cancer cell lines infected with shSETD1A and shGFP. The mutational status of *p53*, *Rb*, *p16* and *K-Ras* in these cell lines is shown below. wt=wild type; mut=mutant. shSETD1Aav represents the mean of cells infected with two different shSETD1A constructs. Data from three independent experiments are presented as Mean±SD; *p<0.05 by two-tailed unpaired Student's t test.

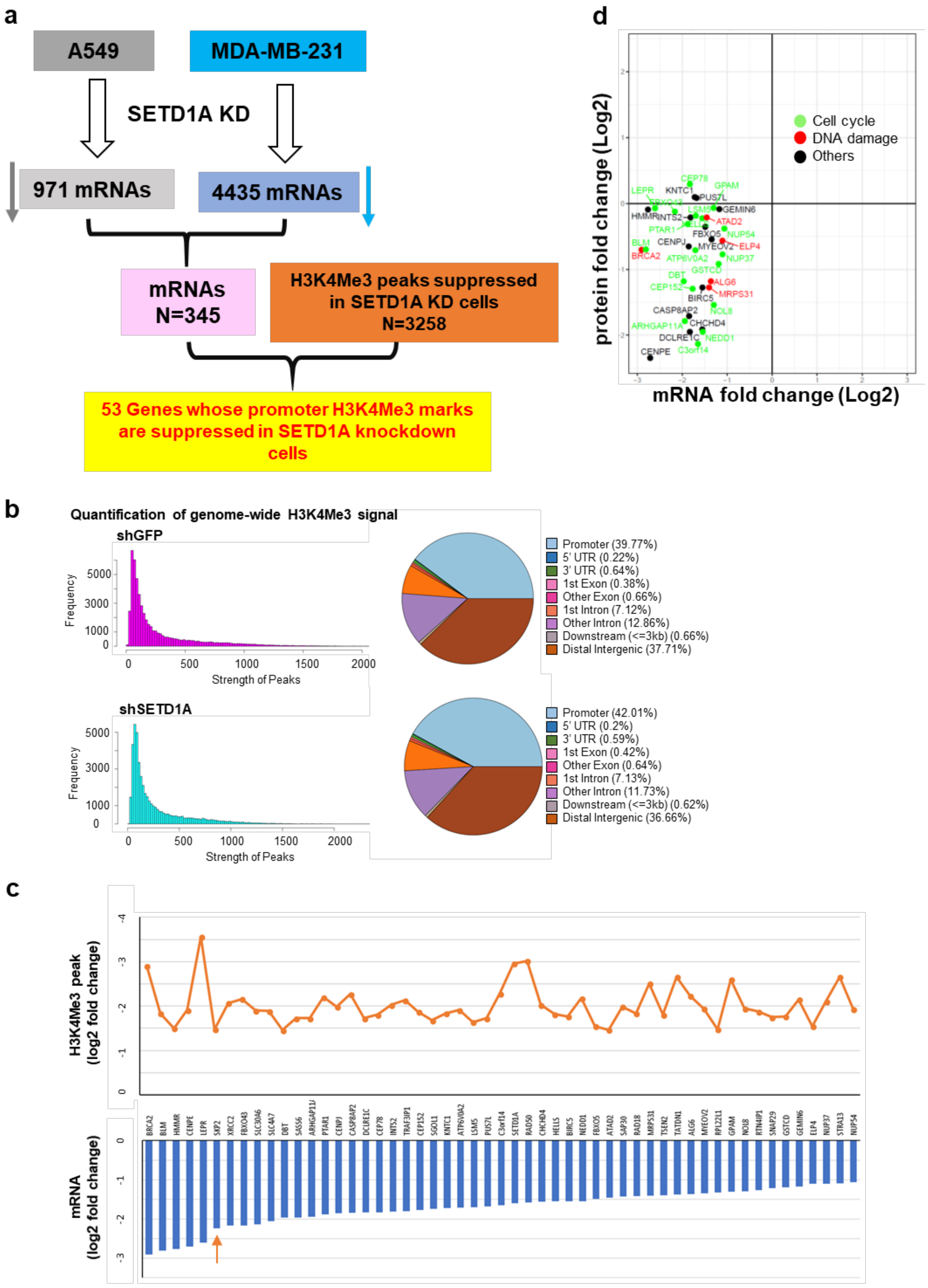
a
A549
MDA-MB-231
SETD1A KD
971 mRNAs
4435 mRNAs
mRNAs N=345
H3K4Me3 peaks suppressed in SETD1A KD cells N=3258
53 Genes whose promoter H3K4Me3 marks are suppressed in SETD1A knockdown cells
b
Quantification of genome-wide H3K4Me3 signal
shGFP
Frequency
Strength of Peaks
Promoter (39.77%)
5' UTR (0.22%)
3' UTR (0.64%)
1st Exon (0.38%)
Other Exon (0.66%)
1st Intron (7.12%)
Other Intron (12.86%)
Downstream (<=3kb) (0.66%)
Distal Intergenic (37.71%)
shSETD1A
Promoter (42.01%)
5' UTR (0.2%)
3' UTR (0.59%)
1st Exon (0.42%)
Other Exon (0.64%)
1st Intron (7.13%)
Other Intron (11.73%)
Downstream (<=3kb) (0.62%)
Distal Intergenic (36.66%)
c
H3K4Me3 peak (log2 fold change)
mRNA (log2 fold change)
d
protein fold change (Log2)
mRNA fold change (Log2)
Cell cycle
DNA damage
Others

Supplementary Fig. 3. SETD1A regulates mitosis, cell cycle and DNA damage response genes

3a: Schema showing the identification of 53 direct SETD1A targets. Transcriptome analysis of A549 and MDA-MB-231 cells following SETD1A-KD (with two different shSETD1A constructs) compared with shGFP expressing cells identifies 971 and 4435 mRNAs suppressed by 0.5-fold in SETD1A-depleted A549 and MDA-MB-231 cells, respectively. Comparison of these transcripts identifies 345 mRNAs which are commonly suppressed in both cell lines. ChIP-Sequencing analysis was used to identify H3K4Me3 peaks suppressed in MDA-MB-231 cells following SETD1A-KD. shGFP-infected cells were used as control. N=3 experimental replicates. Comparison of the H3K4Me3 marks significantly suppressed on 3258 promoters in SETD1A-KD cells with the 345 mRNAs commonly suppressed in both A549 and MDA-MB-231 cells identifies 53 direct targets.

3b: Quantification of the H3K4Me3 marks in both shGFP- and shSETD1A-MDA-MB-231 cells shows the percentage attributed to the various regions across the genome.

3c: Upper: Log2 fold change in the H3K4Me3 marks on the promoters of the 53 SETD1A target genes compared to control cells. Lower: Bar graph shows the log2 fold change in these 53 mRNAs compared with the shGFP control cells. SKP2 is marked with an arrow. Source data are provided as a Source Data file.

3d: The plots show the fold change in RNA and protein of the 53 SETD1A targets in SETD1A-KD cells. Of the 53 direct SETD1A mRNA targets identified, only 36 proteins were detected by mass spectrometry. Of those 36 proteins, 92% (N=33) were suppressed at the protein level. Gene sets are colored based on functional association and gene names are provided (cell cycle/mitosis=green; DNA damage response=red).

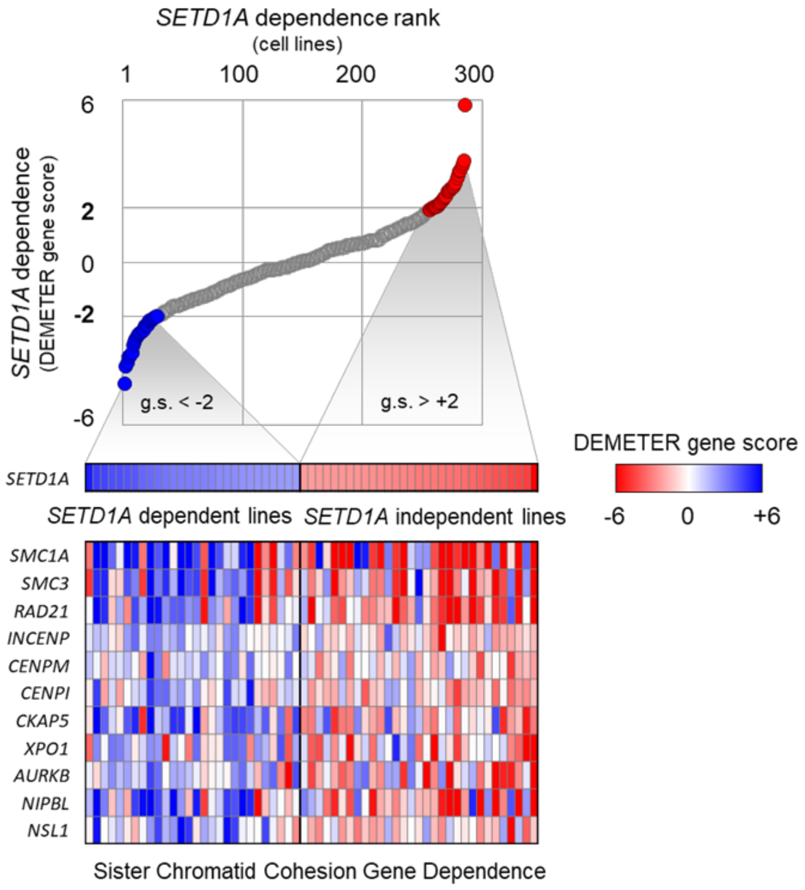
SETD1A dependence rank
(cell lines)
1
100
200
300
6
2
0
-2
-6
SETD1A dependence
(DEMETER gene score)
g.s. < -2
g.s. > +2
DEMETER gene score
SETD1A
SETD1A dependent lines
SETD1A independent lines
-6
0
+6
SMC1A
SMC3
RAD21
INCENP
CENPM
CENPI
CKAP5
XPO1
AURKB
NIPBL
NSL1
Sister Chromatid Cohesion Gene Dependence

b

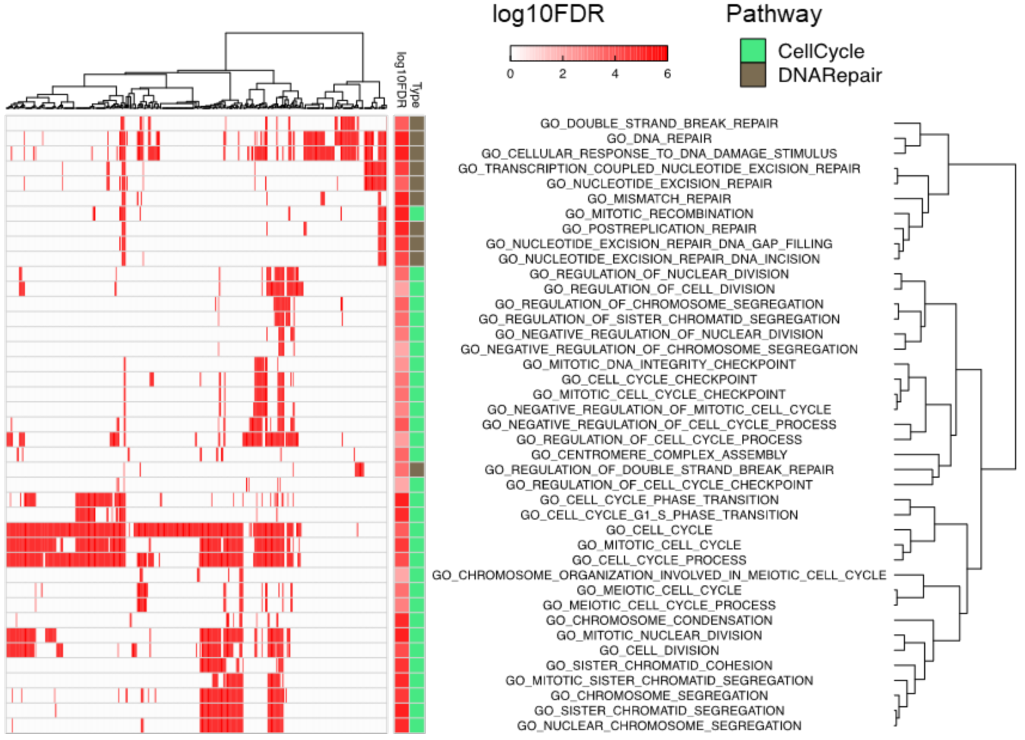
log10FDR
Pathway
0
2
4
6
CellCycle
DNARepair
log10FDR
Type
GO_DOUBLE_STRAND_BREAK_REPAIR
GO_DNA_REPAIR
GO_CELLULAR_RESPONSE_TO_DNA_DAMAGE_STIMULUS
GO_TRANSCRIPTION_COUPLED_NUCLEOTIDE_EXCISION_REPAIR
GO_NUCLEOTIDE_EXCISION_REPAIR
GO_MISMATCH_REPAIR
GO_MITOTIC_RECOMBINATION
GO_POSTREPLICATION_REPAIR
GO_NUCLEOTIDE_EXCISION_REPAIR_DNA_GAP_FILLING
GO_NUCLEOTIDE_EXCISION_REPAIR_DNA_INCISION
GO_REGULATION_OF_NUCLEAR_DIVISION
GO_REGULATION_OF_CELL_DIVISION
GO_REGULATION_OF_CHROMOSOME_SEGREGATION
GO_REGULATION_OF_SISTER_CHROMATID_SEGREGATION
GO_NEGATIVE_REGULATION_OF_NUCLEAR_DIVISION
GO_NEGATIVE_REGULATION_OF_CHROMOSOME_SEGREGATION
GO_MITOTIC_DNA_INTEGRITY_CHECKPOINT
GO_CELL_CYCLE_CHECKPOINT
GO_MITOTIC_CELL_CYCLE_CHECKPOINT
GO_NEGATIVE_REGULATION_OF_MITOTIC_CELL_CYCLE
GO_NEGATIVE_REGULATION_OF_CELL_CYCLE_PROCESS
GO_REGULATION_OF_CELL_CYCLE_PROCESS
GO_CENTROMERE_COMPLEX_ASSEMBLY
GO_REGULATION_OF_DOUBLE_STRAND_BREAK_REPAIR
GO_REGULATION_OF_CELL_CYCLE_CHECKPOINT
GO_CELL_CYCLE_PHASE_TRANSITION
GO_CELL_CYCLE_G1_S_PHASE_TRANSITION
GO_CELL_CYCLE
GO_MITOTIC_CELL_CYCLE
GO_CELL_CYCLE_PROCESS
GO_CHROMOSOME_ORGANIZATION_INVOLVED_IN_MEIOTIC_CELL_CYCLE
GO_MEIOTIC_CELL_CYCLE
GO_MEIOTIC_CELL_CYCLE_PROCESS
GO_CHROMOSOME_CONDENSATION
GO_MITOTIC_NUCLEAR_DIVISION
GO_CELL_DIVISION
GO_SISTER_CHROMATID_COHESION
GO_MITOTIC_SISTER_CHROMATID_SEGREGATION
GO_CHROMOSOME_SEGREGATION
GO_SISTER_CHROMATID_SEGREGATION
GO_NUCLEAR_CHROMOSOME_SEGREGATION

Supplementary Fig. 4. Direct visualization of correlation between dependence on genes that mediate sister chromatid cohesion and SETD1A dependence

4a: Upper: Achilles cell lines are ranked by SETD1A gene score, from most to least dependent. Gene score vs. rank is plotted for each line (gray), and SETD1A-dependent (blue, gene score < -2) and independent lines (red, gene score > +2) are highlighted. Lower: Dependence on genes involved in sister chromatid cohesion, identified by GO gene set enrichment (Fig. 3b), is shown. DEMETER gene scores range from blue (negative, most dependent) to red (positive, least dependent).

4b: Analysis of high-throughput quantitative proteome data available from a panel of 41 breast cancer cell lines shows that endogenous baseline SETD1A protein expression significantly correlates with pathways involving mitosis, cell cycle and DNA damage responses. The heatmap displays the hierarchical clustering of the leading-edge proteins related to the processes indicated; the key to the FDR values is also provided. The full set of gene enrichment results are provided in the Supplementary table 1.

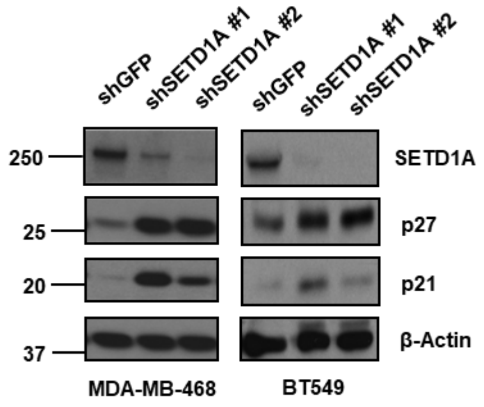

Supplementary Fig. 5. SETD1A-KD in *RB and p53* mutant cells induces p21 and p27
Western blot shows the induction of p21 and p27 proteins in SETD1A-KD MDA-MB-468 and BT549 cells, in which both *p53* and *RB* are inactivated. ß-actin is shown as control. Source data are provided as a Source Data file.

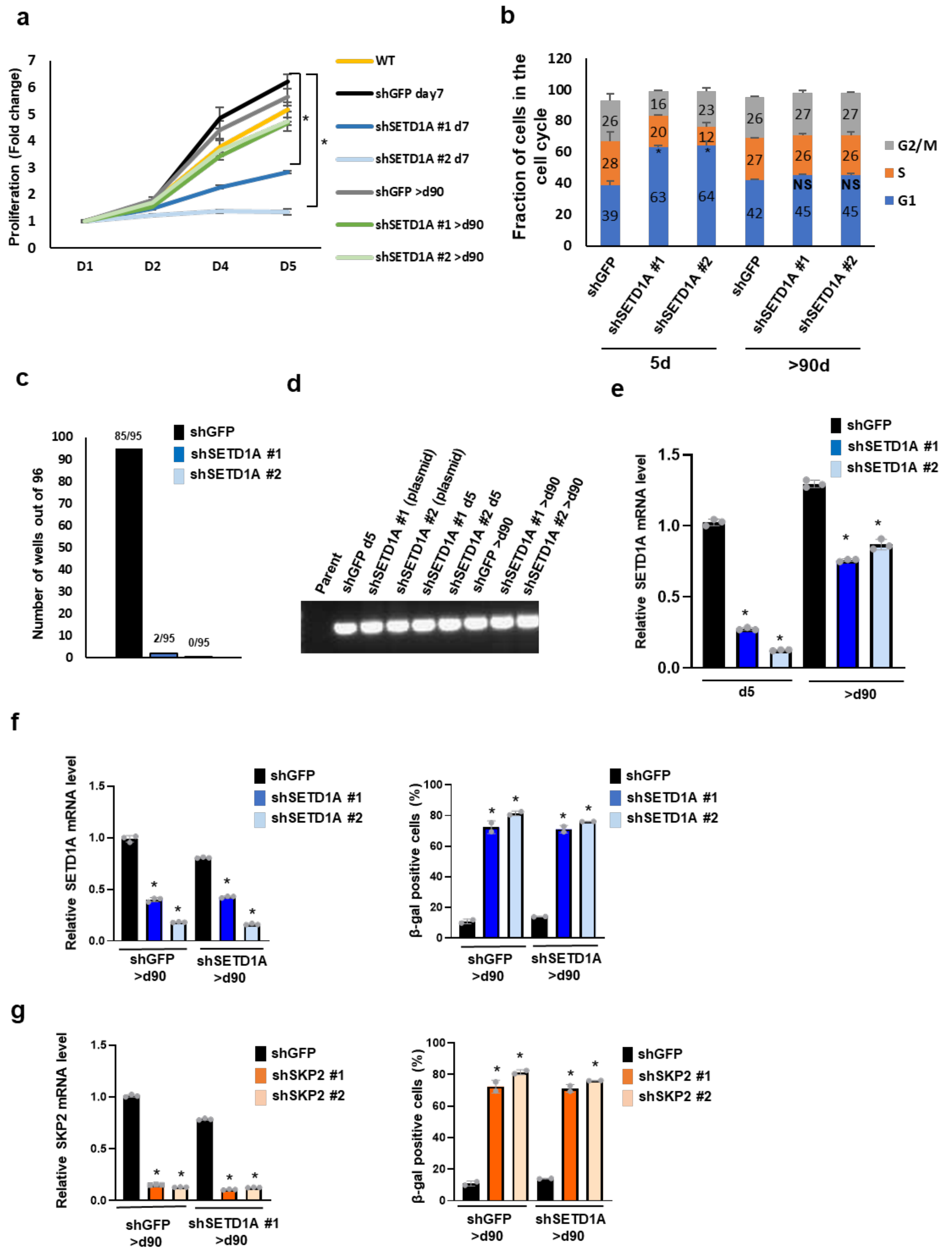
a
Proliferation (Fold change)
D1
D2
D4
D5
WT
shGFP day7
shSETD1A #1 d7
shSETD1A #2 d7
shGFP >d90
shSETD1A #1 >d90
shSETD1A #2 >d90
b
Fraction of cells in the cell cycle
G2/M
S
G1
shGFP
shSETD1A #1
shSETD1A #2
5d
>90d
NS
c
Number of wells out of 96
85/95
2/95
0/95
shGFP
shSETD1A #1
shSETD1A #2
d
Parent
shGFP d5
shSETD1A #1 (plasmid)
shSETD1A #2 (plasmid)
shSETD1A #1 d5
shSETD1A #2 d5
shGFP >d90
shSETD1A #1 >d90
shSETD1A #2 >d90
e
Relative SETD1A mRNA level
d5
>d90
f
Relative SETD1A mRNA level
β-gal positive cells (%)
shGFP >d90
shSETD1A >d90
g
Relative SKP2 mRNA level
shSKP2 #1
shSKP2 #2
shSETD1A #1 >d90

Supplementary Fig. 6. SETD1A-KD cells escape senescence

6a: Proliferation of the SETD1A-KD senescent (5 days after infection) and senescence-escaping cultures (>90d) is shown. Data from three independent experiments are presented as Mean±SD; *$p<0.05$ by two-tailed unpaired Student's t test. Source data are provided as a Source Data file.

6b: Cell cycle analysis of SETD1A-KD cells maintained for >90 days in culture shows re-entry into the cell cycle (Escape) compared with the cell cycle arrest exhibited by SETD1A-KD senescent cells (5 days after SETD1A-KD). Quantification of cells at each stage of the cell cycle. Data from three independent experiments are presented as Mean±SD; *$p<0.05$ by two-tailed unpaired Student's t test. Source data are provided as a Source Data file.

6c: Individual SETD1A-KD (n=190; 95 individual cells from each of shSETD1A#1 and shSETD1A#2 cultures) and control cells (n=95) were seeded into 96-well plates and the number of colonies were scored. Source data are provided as a Source Data file.

6d: Genomic DNA from control (shGFP) and shSETD1A cells that are senescent or have escaped senescence was analyzed by PCR for the integration of the PLKO plasmid. Water was used as negative control and the plasmids were used as positive controls.

6e: Quantification of relative SETD1A mRNA levels (Mean±SD) in ShSETD1A-KD senescent and escape cultures. shGFP cultures are shown as controls. Data from three independent experiments are presented as Mean±SD; *$p<0.05$ by two-tailed unpaired Student's t test. Source data are provided as a Source Data file.

6f: Knockdown of SETD1A in SETD1A-KD senescence escaping cells induces senescence. Left: Quantification of relative SETD1A mRNA levels (Mean±SD) in shSETD1A-KD escape cells following knockdown of SETD1A with two different shSETD1A constructs. Matched shGFP cultures are shown as controls. Data from two independent experiments are presented as Mean±SD; *$p<0.05$ by two-tailed unpaired Student's t test. Right: Percentage of ß-gal positive cells in each of the samples shown on the left. Data from two independent experiments are presented as Mean±SD; *$p<0.05$ by two-tailed unpaired Student's t test. Source data are provided as a Source Data file.

6g: Knockdown of SKP2 in the SETD1A-KD senescence-escaping cells induces senescence. Left: Quantification of relative SKP2 mRNA levels (Mean±SD) in shSETD1A-KD escape cells following SKP2-KD with two different shSKP2 constructs. The matched shGFP cultures are shown as controls. Data from two to three independent experiments are presented as Mean±SD; *$p<0.05$ by two-tailed unpaired Student's t test. Source data are provided as a Source Data file. Right: Percentage of ß-gal positive cells in each of the samples shown on the left. Data from two to three independent experiments are presented as Mean±SD; *$p<0.05$ by two-tailed unpaired Student's t test. Source data are provided as a Source Data file.

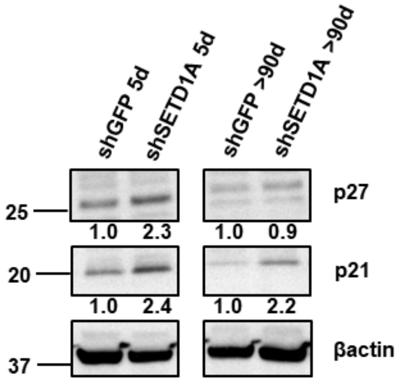

Supplementary Fig. 7. p21 and p27 expression in SETD1A-KD senescence escaping cells. Appropriate shGFP cells are shown as controls and ß-actin is used as loading control. Quantification of the bands is provided below each blot. Source data are provided as a Source Data file.

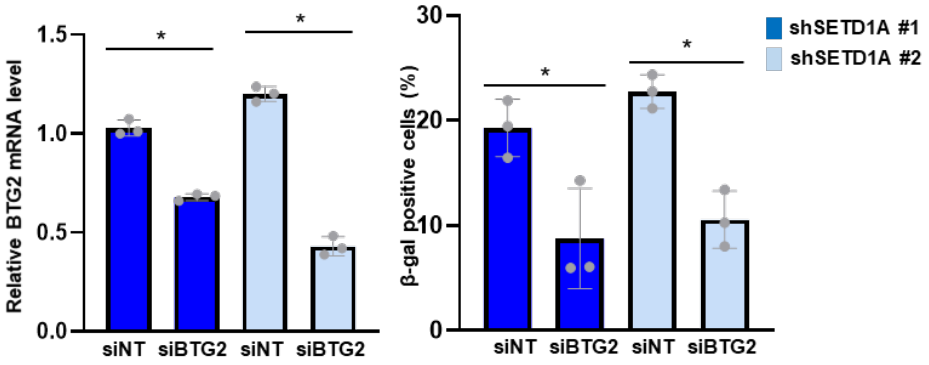

Supplementary Fig. 8. BTG2 contributes to SETD1A-mediated senescence
Knockdown of BTG2 rescues the senescence phenotype. SETD1A expression was knocked down in cells following the suppression of BTG2 (using siRNAs) and the ß-Gal positive cells were enumerated. Non-targeting siRNA was used as control. Left: Bar graph shows the relative BTG2 mRNA levels in SETD1A-KD cells (performed with two different shRNAs against SETD1A) transfected with siRNAs against BTG2. Data from three independent experiments are presented as Mean±SD; *p<0.05 by two-tailed unpaired Student's t test. Right: Bar graph shows the percentage of ß-gal positive cells in the SETD1A-KD cells shown on the left. Data from three independent experiments are presented as Mean±SD; *p<0.05 by two-tailed unpaired Student's t test. Source data are provided as a Source Data file.

Supplementary Table 1
Description of the leading-edge proteins related to the processes shown in Fig. S3B

#	Signatures	GO
1	Centrosome	GO:0005813
2	Nucleoplasm	GO:0005654
3	Cell division	GO:0051301
4	G2/M transition of mitotic cell cycle	GO:0000086
5	Condensed chromosome kinetochore	GO:0000777
6	Mitotic nuclear division	GO:0007067
7	Strand displacement	GO:0000732
8	DNA synthesis involved in DNA repair	GO:0000731
9	Sister chromatid cohesion	GO:0007062
10	Centriole	GO:0005814
11	Chromosome, centromeric region	GO:0000775
12	Chromosome	GO:0005694
13	Chromosome organization	GO:0051276
14	Poly A RNA binding	GO:0003723
15	RNA binding	GO:0003723
16	Sister chromatid segregation	GO:0000819
17	Nuclear chromosome segregation	GO:0098813
18	Negative regulation of nitrogen compound metabolic process	GO:0051172
19	Cell cycle process	GO:0022402
20	Double strand break repair	GO:0006302
21	DNA repair	GO:0045021
22	Cellular response to DNA damage stimulus	GO:2001020
23	Transcription coupled nucleotide excision repair	GO:0006283
24	Nucleotide excision repair	GO:0006289
25	Mitotic recombination	GO:0006312
26	Post replication repair	GO:0006301
27	Nucleotide excision repair DNA gap filling	GO:0006297
28	Nucleotide excision repair DNA incision	GO:0033683
29	Regulation of nuclear division	GO:0051783
30	Regulation of cell division	GO:0051302
31	Regulation of chromosome segregation	GO:0051983
32	Regulation of sister chromatid segregation	GO:0033045

33	Negative regulation of chromosome segregation	GO:0051985
34	Mitotic DNA integrity checkpoint	GO:0044774
35	Cell cycle check point	GO:0000075
36	Negative regulation of mitotic cell cycle	GO:0045930
37	Negative regulation of cell cycle process	GO:0010948
38	Regulation of cell cycle process	GO:0010564
39	Centromere complex assembly	GO:0034508
40	Regulation of double strand break repair	GO:2000779
41	Regulation of cell cycle check point	GO:1901976
42	Cell cycle phase transition	GO:0044770
43	Cell cycle G1/S phase transition	GO:0044843
44	Cell cycle	GO:0007049
45	Mitotic cell cycle	GO:0000278
46	Cell cycle process	GO:0022402
47	Chromosome organization involved in meiotic cell cycle	GO:0070192
48	Meiotic cell cycle	GO:0051321
49	Meiotic cell cycle process	GO:1903046
50	Chromosome condensation	GO:0030261
51	Mitotic nuclear division	GO:0140014
52	Cell division	GO:0051301
53	Sister chromatid cohesion	GO:0051177
54	Mitotic sister chromatid segregation	GO:0000070
55	Chromosomal segregation	GO:0007059
56	Sister chromatid segregation	GO:0000819
57	Nuclear chromosome segregation	GO:0098813
