# Supplementary material for: SETD1A protects from senescence through regulation of the mitotic gene expression program
Source: Nat Commun. 2019 Jun 28;10:2854. doi: 10.1038/s41467-019-10786-w (PMC6599037; doi:10.1038/s41467-019-10786-w)
Supplement: Supplementary file 2 — Reporting Summary [file 41467_2019_10786_MOESM2_ESM.pdf]

natureresearch

Corresponding author(s): Shyamala Maheswaran

Last updated by author(s): Feb 14, 2019

Reporting Summary

Nature Research wishes to improve the reproducibility of the work that we publish. This form provides structure for consistency and transparency in reporting. For further information on Nature Research policies, see Authors & Referees and the Editorial Policy Checklist.

Statistics

For all statistical analyses, confirm that the following items are present in the figure legend, table legend, main text, or Methods section.

n/a	Confirmed	
☐	☒	The exact sample size (*n*) for each experimental group/condition, given as a discrete number and unit of measurement
☐	☒	A statement on whether measurements were taken from distinct samples or whether the same sample was measured repeatedly
☐	☒	The statistical test(s) used AND whether they are one- or two-sided<br>*Only common tests should be described solely by name; describe more complex techniques in the Methods section.*
☐	☒	A description of all covariates tested
☐	☒	A description of any assumptions or corrections, such as tests of normality and adjustment for multiple comparisons
☐	☒	A full description of the statistical parameters including central tendency (e.g. means) or other basic estimates (e.g. regression coefficient) AND variation (e.g. standard deviation) or associated estimates of uncertainty (e.g. confidence intervals)
☐	☒	For null hypothesis testing, the test statistic (e.g. *F*, *t*, *r*) with confidence intervals, effect sizes, degrees of freedom and *P* value noted<br>*Give P values as exact values whenever suitable.*
☒	☐	For Bayesian analysis, information on the choice of priors and Markov chain Monte Carlo settings
☒	☐	For hierarchical and complex designs, identification of the appropriate level for tests and full reporting of outcomes
☐	☒	Estimates of effect sizes (e.g. Cohen's *d*, Pearson's *r*), indicating how they were calculated

Our web collection on statistics for biologists contains articles on many of the points above.

Software and code

Policy information about availability of computer code

Data collection	FACS data was collected using FlowJo(v10), Mass Spectrometry data collected by Thermo Fisher Tune and Xcalibur software in conjunction with a SEQUEST related analysis platform provided by the Gygi Lab.
Data analysis	FlowJo(v10) was used for FACS analysis. ELK was used for MassSpec analysis. Survival analysis was done using the KM plotter web server. Cytoscape (v3.5.1) and STRING (v10.5) were used to annotate and visualize protein-protein interactions. ChIP-seq SOLiD reads were analyzed using the LifeScope (v2.5) software package. BAM files were subjected to peak calling using MACS2, which generated narrowPeak files. R/Bioconductor packages "ChIPseeker" and "clusterProfiler" were used to annotate peak sites near known human genes. Functional enrichments were generated using the DAVID webserver. Geneset enrichment analysis for Biological process GO terms was performed using the BROAD pre-ranked GSEA tool (v2.2.2). Heatmaps were generated using R. T-tests were performed using Excel or SPSS.

For manuscripts utilizing custom algorithms or software that are central to the research but not yet described in published literature, software must be made available to editors/reviewers. We strongly encourage code deposition in a community repository (e.g. GitHub). See the Nature Research guidelines for submitting code & software for further information.

Data

Policy information about availability of data

All manuscripts must include a data availability statement. This statement should provide the following information, where applicable:

- Accession codes, unique identifiers, or web links for publicly available datasets
- A list of figures that have associated raw data
- A description of any restrictions on data availability

ChIP sequencing data have been deposited in the NCBI
Gene Expression Omnibus database under accession code
GSE 117427.
Microarray data have been deposited in the NCBI Gene

Expression Omnibus database under accession code GSE 71498.
All relevant data are available from the authors.

Field-specific reporting

Please select the one below that is the best fit for your research. If you are not sure, read the appropriate sections before making your selection.

☒ Life sciences ☐ Behavioural & social sciences ☐ Ecological, evolutionary & environmental sciences

For a reference copy of the document with all sections, see nature.com/documents/nr-reporting-summary-flat.pdf

Life sciences study design

All studies must disclose on these points even when the disclosure is negative.

Sample size	No sample size calculation was performed.
Data exclusions	No data was excluded from analysis.
Replication	All attempts at replication was successful.
Randomization	No randomization was used.
Blinding	No blind method was used.

Reporting for specific materials, systems and methods

We require information from authors about some types of materials, experimental systems and methods used in many studies. Here, indicate whether each material, system or method listed is relevant to your study. If you are not sure if a list item applies to your research, read the appropriate section before selecting a response.

Materials & experimental systems

n/a	Involved in the study	
☐	☒	Antibodies
☐	☒	Eukaryotic cell lines
☒	☐	Palaeontology
☒	☐	Animals and other organisms
☒	☐	Human research participants
☒	☐	Clinical data

Methods

n/a	Involved in the study	
☐	☒	ChIP-seq
☐	☒	Flow cytometry
☒	☐	MRI-based neuroimaging

Antibodies

Antibodies used	Antibodies used were rabbit anti-SETD1A polyclonal antibody (NOVUS) , rabbit anti-p21 polyclonal antibody (C-19, Santa Cruz), rabbit anti-p27 polyclonal antibody (C-19, Santa Cruz), rabbit anti-cleaved caspase-3 polyclonal antibody (Santa Cruz), rabbit anti-PARP antibody (Cell Signaling), rabbit anti-Histone H3 antibody (Abcam), rabbit anti-SKP2 antibody (Cell Signaling), rabbit anti-Histone H3 mono-methyl K4 antibody (Abcam), Goat anti-Histone H3 di-methyl K4 antibody (Abcam), rabbit anti-Histone H3 tri-methyl K4 antibody (Abcam) and mouse anti-Actin monoclonal antibody (BD Bioscience).
Validation	Antibody validations were performed by antibody suppliers per quality assurance literature provided by each supplier.

Eukaryotic cell lines

Policy information about cell lines

Cell line source(s)	All cells lines were acquired from ATCC.
Authentication	All cell lines were periodically authenticated and were matched with the earliest passage cell lines.
Mycoplasma contamination	Cell lines were periodically tested for mycoplasma contamination and shown as negative for infection.
Commonly misidentified lines (See ICLAC register)	No commonly misidentified lines were used in this study.

ChIP-seq

Data deposition

☒ Confirm that both raw and final processed data have been deposited in a public database such as GEO.

☒ Confirm that you have deposited or provided access to graph files (e.g. BED files) for the called peaks.

Data access links<br>*May remain private before publication.*	https://www.ncbi.nlm.nih.gov/geo/query/acc.cgi?acc=GSE117427<br>reviewer token: uhsfqyactxyhnan
Files in database submission	BAM files: shGFP_H3K4me3_1.bam<br>shGFP_H3K4me3_2.bam<br>shGFP_H3K4me3_3.bam<br>shSETD1A_H3K4me3_1.bam<br>shSETD1A_H3K4me3_2.bam<br>shSETD1A_H3K4me3_3.bam<br>NarrowPeak files:shGFP_H3K4me3_1_peaks.narrowPeak<br>shGFP_H3K4me3_2_peaks.narrowPeak<br>shGFP_H3K4me3_3_peaks.narrowPeak<br>shSETD1A_H3K4me3_1_peaks.narrowPeak<br>shSETD1A_H3K4me3_2_peaks.narrowPeak<br>shSETD1A_H3K4me3_3_peaks.narrowPeak
Genome browser session<br>(e.g. UCSC)	*Provide a link to an anonymized genome browser session for "Initial submission" and "Revised version" documents only, to enable peer review. Write "no longer applicable" for "Final submission" documents.*

Methodology

Replicates	3
Sequencing depth	All reads are 50bp single-end. Below are the total read and uniquely mapped reads for each BAM file.<br>shGFP_H3K4me3_1.bam Total = ; Unique = 14355019<br>shGFP_H3K4me3_2.bam Total = ; Unique = 13811050<br>shGFP_H3K4me3_3.bam Total = ; Unique = 15886035<br>shSETD1A_H3K4me3_1.bam Total = ; Unique = 12140155<br>shSETD1A_H3K4me3_2.bam Total = ; Unique =15633379<br>shSETD1A_H3K4me3_3.bam Total = ; Unique =15415484
Antibodies	rabbit anti-Histone H3 mono-methyl K4 antibody (Abcam)
Peak calling parameters	The mapping software (ABI LifeScope) used default parameters. It takes XQC and generate UNIQUELY mapped BAMS. Peaks were called using MACS2 the following parameters: macs2 callpeak -t ChIP.bam -c Control.bam -f BAM -g hs -n test -q 0.01.
Data quality	The alignment tool used the default quality control thresholds and provided only uniquely mapped reads as output in the BAM files. The MACS2 peak caller used a stringent cutoff of FDR 1% for detection of peaks. The following lists provide the counts of peak with FDR < 1% and peaks with Fold change > 5 for the comparison of shSETD1A samples against shGFP control samples.<br>shSETD1A_H3K4me3_1: FDR peaks = 27901; fold change peaks = 22638<br>shSETD1A_H3K4me3_2: FDR peaks = 31122; fold change peaks = 23695<br>shSETD1A_H3K4me3_3: FDR peaks = 32300; fold change peaks = 24654
Software	XSQ colour space read files were subjected to analyses using LifeScope software package (Life Technologies), which provided a ChIP-seq module. LifeScope aligned color space read data to the hg19 human genome reference sequence to generate BAM files.<br>BAM files were subjected to peak calling using MACS2, which generated .narrowPeak files.<br>R/Bioconductor packages "ChIPseeker" and "clusterProfiler" were used to perform tertiary analyses.

Flow Cytometry

Plots

Confirm that:

☒ The axis labels state the marker and fluorochrome used (e.g. CD4-FITC).

☒ The axis scales are clearly visible. Include numbers along axes only for bottom left plot of group (a 'group' is an analysis of identical markers).

☐ All plots are contour plots with outliers or pseudocolor plots.

☒ A numerical value for number of cells or percentage (with statistics) is provided.

Methodology

Sample preparation	Cells were harvested, washed twice in PBS, resuspended in 70% ethanol overnight, and then diluted in propidium iodide, RNase staining buffer (BD Pharmingen) and incubated for 15 min at 37oC.
Instrument	Flow cytometric analysis was performed on a FORTESSA Cell analyzer (Cat No.647177).
Software	FloJo (V10) was used for flow cytometry data analysis.
Cell population abundance	Before cells were harvested, we confirmed all cells were viable. In the sorting, a viable cells gate were defined from FSC-A v SSC-A.
Gating strategy	A viable cell gate was defined first from FSC-A v SSC-A. Singlet gates were then defined on FSC-H v FSC-W. Finally, the DNA contents were quantified with propidium iodide Dye.

☐ Tick this box to confirm that a figure exemplifying the gating strategy is provided in the Supplementary Information.
