# Supplementary material for: SETD1A protects from senescence through regulation of the mitotic gene expression program
Source: Nat Commun. 2019 Jun 28;10:2854. doi: 10.1038/s41467-019-10786-w (PMC6599037; doi:10.1038/s41467-019-10786-w)
Supplement: Supplementary file 3 — Description of Additional Supplementary Files [file 41467_2019_10786_MOESM3_ESM.pdf]

Description of Additional Supplementary Files

File name: Supplementary Movie 1
Description: Live-imaging of RFP-tagged H2B expressing A549 cells undergoing senescence. Imaging was begun ~24 hours after lentiviral infection with shGFP constructs. These control cells show proper mitosis. Time is shown in hr:min.

File name: Supplementary Movie 2
Description: Live-imaging of RFP-tagged H2B expressing A549 cells. Imaging was begun ~24 hours after lentiviral infection of the shSETD1A construct. SETD1A-KD cells show mitotic defects preceding the senescence phenotype. Time is shown in hr:min.
